# *Toxoplasma gondii* assembles extracellular vesicles with conserved lipid profiles across host cell types

**DOI:** 10.3389/fcimb.2026.1745625

**Published:** 2026-02-04

**Authors:** Teresa Cruz-Bustos, Anja Joachim

**Affiliations:** Institute of Parasitology, Department of Biological Sciences and Pathobiology, University of Veterinary Medicine Vienna, Vienna, Austria

**Keywords:** apicomplexa, exovesicles, host-parasite interaction, lipidome, mass-spectrometric analysis

## Abstract

**Introduction:**

*Toxoplasma gondii* is an obligate intracellular parasite with an exceptional capacity to colonize a broad range of host species and cell types. Successful infection depends on its ability to manipulate host metabolism, including lipid pathways that are essential for membrane biogenesis, signalling, and immune modulation. Extracellular vesicles (EVs) are increasingly recognized as critical mediators of parasite–host interactions, but while their protein and nucleic acid cargo has been studied, the lipid composition of T. gondii EVs (TgEVs) remains poorly defined.

**Methods:**

In this study, we performed a lipidomic analysis of TgEVs secreted by tachyzoites grown in four distinct host cell types: fibroblasts, Vero cells, myoblasts, and porcine intestinal epithelial cells (IPEC). Cells and TgEVs were isolated from five biological replicates per condition and analysed by liquid chromatography coupled to high-resolution tandem mass spectrometry. Comparative lipid profiling of TgEVs and their corresponding host cells was performed after total ion current normalization, followed by principal component analysis to capture global compositional patterns and pairwise differential abundance testing to identify significantly enriched or depleted lipid species.

**Results:**

We identified 194 lipid species across 15 classes. Despite metabolic differences among host cell types, TgEVs displayed a highly conserved and distinctive lipid profile. Glycerophospholipids such as phosphatidylcholine (PC) and phosphatidylethanolamine (PE) were the most abundant components, while sphingolipids, including sphingomyelin and ceramides, were consistently present and likely contribute to vesicle biogenesis and cargo organization. Notably, triacylglycerols (TG) were enriched in TgEVs across all host conditions, suggesting active selection of neutral lipids during vesicle formation. Correlation analyses confirmed that TgEV lipidomes diverge from their cellular origin, indicating a process of active sorting rather than passive acquisition from the host membrane.

**Discussion:**

These findings indicate that T. gondii produces vesicles with conserved and distinctive lipid compositions that differ from those of the host cell. This selective lipid core hints at key functions in parasite, host communication, immune modulation, nutrient acquisition, and other vesicle–cell interactions. Our work advances the molecular understanding of TgEVs and establishes a foundation for future studies into how lipid-mediated signalling contributes to the complex dynamics of T. gondii infections in different cellular environments.

## 1 Introduction

*Toxoplasma gondii* is an obligate intracellular protozoan that infects nearly all warm-blooded animals, including humans, making it a pathogen of major public health and veterinary concern (Dubey, 2023). As the causative agent of toxoplasmosis, *T. gondii* disproportionately affects immunocompromised individuals and can cause severe congenital complications during pregnancy (Dubey, 2008). Its success as a parasite relies on its capacity to manipulate host cellular processes to create a suitable environment for growth and replication. To achieve this, *T. gondii* employs multiple molecular strategies, including the secretion of effector proteins that rewire host signalling and the selective exploitation of lipid metabolism pathways for membrane biogenesis and immunomodulation (Joiner and Roos, 2002; Laliberté and Carruthers, 2008). A critical axis of this host-parasite interaction is lipid metabolism. *T. gondii* employs a composite lipid acquisition strategy, relying on both *de novo* synthesis in its apicoplast and the active scavenging of essential host lipids, such as cholesterol and sphingolipids (Coppens et al., 2000). This metabolic plasticity allows it to thrive in the distinct lipid environments of different tissues, directly influencing its replication dynamics, organelle biogenesis, and virulence (Walsh et al., 2022; He et al., 2024). The parasite significantly rewires host lipid metabolism, manipulating lipid droplets and subverting trafficking pathways to support the development of the parasitophorous vacuole and the parasite’s rapid growth (Xu et al., 2020; Shunmugam et al., 2022). Lipids play essential and multifaceted roles for both parasite and host during infection (Haase et al., 2023). Beyond serving as structural components of cellular membranes (Muro et al., 2014), lipids regulate signalling pathways (Wymann and Schneiter, 2008), energy storage (Walther and Farese, 2012), organelle dynamics (Domingues et al., 2025), and they also predict a hybrid metabolic origin of *T. gondii* EVs (TgEVs), with potential implications for immune evasion and tissue tropism immune responses (Torres and Cyster, 2023). Despite these fundamental roles, the full diversity of lipid functions during *T. gondii* infection remains incompletely understood, particularly with regard to how the parasite exports lipid species to shape host-pathogen interactions.

An emerging and crucial mechanism in these complex interactions is the secretion of extracellular vesicles (EVs) (Sibley et al., 1986; Silva et al., 2018; Quiarim et al., 2021; Ramírez-Flores et al., 2019). EVs are lipid bilayer–enclosed particles secreted by cells, playing fundamental roles in communication across all living systems. Based on their size, biogenesis, and molecular composition, EVs are generally categorized into two main subtypes: small EVs (typically <200 nm), which originate from the endocytic pathway and are released upon fusion of multivesicular bodies with the plasma membrane, and large EVs (0.2–1 µm), which bud directly from the plasma membrane (Welsh et al., 2024). Both subtypes mediate the transfer of bioactive cargo, including proteins, lipids, mRNAs, microRNAs (miRNAs), and other non-coding RNAs, thereby influencing the behaviour and function of recipient cells (Fernandez-Becerra et al., 2023; Colombo et al., 2014). EVs are known to regulate a wide range of physiological and pathological processes (Tkach and Théry, 2016). Importantly, EV-mediated signalling operates independently of direct cell–cell contact, enabling communication across both short and long distances (van Niel et al., 2022). In *T. gondii*, a growing body of evidence confirms that TgEVs are loaded with a rich proteomic cargo, including virulence factors and immunomodulatory molecules, which can alter host cell responses and facilitate infection (Wowk et al., 2017; Quiarim et al., 2021; Cruz-Bustos et al., 2025).

However, while the protein and nucleic acid content of TgEVs has garnered increasing attention, their lipid composition remains a profound and largely uncharacterized frontier. This knowledge gap is critical, as lipids are far from passive structural scaffolds; rather, they are dynamic regulators that shape every stage of EV biology, from membrane budding and cargo selection to vesicle release, stability, biodistribution, and interactions with recipient cells (Skotland et al., 2020; Harayama and Riezman, 2018; Fyfe et al., 2023). The physical properties, biological functions, and even the fate of EVs are inextricably linked to their lipid makeup (Brent et al., 2025). Different lipid classes with specific functions contribute to EVs (Skotland et al., 2020; Ghadami and Dellinger, 2023). Glycerophospholipids, such as phosphatidylcholine (PC) and phosphatidylethanolamine (PE), are essential for maintaining membrane integrity, curvature, and fluidity. These parameters directly influence vesicle formation and cargo loading (Hallal et al., 2022). Sphingolipids, including ceramides and glycosphingolipids, are central to the formation of lipid rafts. These microdomains serve as organizational platforms for protein sorting and signalling (van Meer et al., 2008; Hannun and Obeid, 2008). In addition to their structural roles, lipids confer functional bioactivity. For instance, the externalization of phosphatidylserine can enhance EV uptake by immune cells and attenuate inflammatory signalling by mimicking apoptotic cell clearance mechanisms (Hallal et al., 2022), while sphingolipid species have been identified as active mediators of host-pathogen communication (Wang et al., 2021), shaping immune responses and contribute to parasite persistence and virulence. Importantly, neutral lipids such as triacylglycerols (TG), often overlooked in EV studies, may serve additional roles in energy transfer, metabolic signalling, or the packaging of hydrophobic molecules, highlighting the potential multifunctionality of vesicular lipid cargo, Such features suggest that EVs are not merely passive carriers but metabolically active entities capable of influencing the lipid homeostasis and signalling landscape of recipient cells (Skotland et al., 2025).

Given the reliance of *T. gondii* on host-scavenged lipids, we therefore sought to determine whether the TgEV lipidome simply mirrors the composition of host cells or constitutes a unique entity defined by the parasite that remains unchanged across different host environments. In this study, we provide a large-scale lipidomic analysis of EVs secreted by *T. gondii* and their host cell culture. Using high-resolution, mass spectrometry-based approaches, we identified and quantified lipid classes and species within TgEVs and compared their composition to that of host-cell. Our findings offer new insights into the molecular architecture of *T. gondii* EVs and lay the groundwork for future investigations into how EV-associated lipids contribute to parasite survival, host manipulation, and immune evasion.

## 2 Materials and methods

### 2.1 Parasite culture and EV harvesting

The procedures for *T. gondii* culture, extracellular vesicle (EV) isolation and characterisation were conducted as previously described (Cruz-Bustos et al., 2025). In brief, RH strain tachyzoites were propagated in four different host cell lines, human foreskin fibroblasts (Hs27, obtained from ATCC CRL-1634), green monkey kidney epithelial cells (Vero, ATCC CCL-181), mouse myoblasts (C2C12, ATCC CRL-1771) and porcine intestinal epithelial cells (IPEC-1, ACC 705, Leibniz Institute DSMZ-German Collection of Microorganisms and Cell Cultures GmbH, Leibniz, Germany), to isolate EVs produced in diverse cellular environments. Following egress, the parasites were incubated in EV-depleted medium under host cell-free conditions to permit EV release. The EVs were then isolated from the conditioned medium via a series of differential centrifugation steps, including 0.22 µm filtration and ultracentrifugation at 100, 000×*g*. The purity and morphology of the EVs were subsequently assessed using nanoparticle tracking analysis (NTA) and transmission electron microscopy (TEM), in accordance with the MISEV2023 guidelines (Welsh et al., 2024). For the present study, EVs derived from 1 × 10^8^ tachyzoites of *T. gondii* and uninfected host cell cultures were used for lipidomic profiling.

### 2.2 Lipidomic profiling by LC-MS/MS

Lipidomic analysis of TgEVs derived from tachyzoites of *T. gondii* and from uninfected host cells was performed by liquid chromatography coupled to high-resolution tandem mass spectrometry (LC–MS/MS). For the experiment, five biological replicates were analysed. TgEV samples consisted of approximately 1 × 10^9^ extracellular vesicles per biological replicate, while host-cell samples contained 1 × 10^6^ cells per biological replicate.

The TgEVs were dissolved in 100 µL of 90% isopropanol (IPA) containing the internal standards (EquiSPLASH; Avanti Polar Lipids, final concentration 1% v/v), thoroughly vortexed and subsequently centrifuged for 10 min at 15, 000×*g* at 4 °C with a 5415R microcentrifuge (Eppendorf, Hamburg, Germany). After centrifugation, supernatants were transferred to analytical glass vials and placed in the autosampler. For cell lipid extraction, 1 mL of IPA (containing internal standards at the same final concentration as for the TgEVs) were initially added to the cells and homogenized on dry ice with a bead beater (FastPrep-24; MP Biomedicals, CA, USA) at 6.0 m/s (3 x 30 s, 5 min pause time) using 1.0 mm zirconia/glass beads (Biospec Products, OK, USA). After centrifugation for 10 min at 15, 000×*g* and 4 °C. Supernatants were transferred to 1.5mL Eppendorf tubes, dried under a stream of nitrogen, and reconstituted in 100 µL 90% IPA. The final samples were vortexed for 10 min, centrifuged as described above and the supernatants were transferred to analytical glass vials for LC-MS/MS analysis.

Chromatographic separation was achieved using a Vanquish UHPLC system (Thermo Scientific, MA, USA) coupled to an Orbitrap Exploris 240 mass spectrometer (Thermo Scientific), operating in both positive and negative electrospray ionization (ESI) modes. Separation was carried out on an ACQUITY Premier CSH C18 column (2.1 × 100 mm, 1.7 µm; Waters Corporation, Milford, MA, USA) maintained at 65 °C, with a flow rate of 0.3 mL min^−1^. The mobile phases consisted of water:acetonitrile (40:60, v/v; mobile phase A) and isopropanol:acetonitrile (90:10, v/v; mobile phase B), supplemented with 10 mM ammonium acetate and 0.1% acetic acid (negative mode) or 10 mM ammonium formate and 0.1% formic acid (positive mode). The chromatographic gradient (total runtime: 23 min, including re-equilibration) was programmed as follows (%B): 0 min, 15%; 2.5 min, 30%; 3.2 min, 48%; 15 min, 82%; 17.5 min, 99%; 19.5 min, 99%; 20 min, 15%; 23 min, 15%. The autosampler was maintained at 4 °C, and the injection volume was 5 µL. Mass spectrometric acquisition was performed in full-scan mode at a resolving power of 120, 000 over an m/z range of 200–1700 (scan time: 100 ms, RF lens: 70%). Data-dependent MS/MS acquisition was conducted at a resolving power of 15, 000 (scan time: 54 ms) with stepped collision energies of 25, 35, and 50%, and a total cycle time of 600 ms. Ion source parameters were: spray voltage 3250 V (positive)/3000 V (negative), sheath gas 45 psi, auxiliary gas 15 psi, sweep gas 0 psi, ion transfer tube 300 °C, and vaporizer temperature 275 °C.

Samples were analysed in randomized order. Quality control (QC) samples were prepared by pooling equal aliquots of all samples and injected repeatedly at the start of the run to condition the system, and after every fifth sample to monitor analytical performance. Process blanks were included for background assessment and subsequent subtraction. Raw data were processed in MS-DIAL (Tsugawa et al., 2015). Peak intensities were normalized to the total ion current (TIC) of all detected analytes (Drotleff and Lämmerhofer, 2019). Lipid features were annotated based on accurate mass, isotopic distribution, MS/MS fragmentation spectra, retention time, and class-specific elution patterns (Drotleff et al., 2020). Lipidomics data: Raw LC–MS/MS files and LipidSearch result spreadsheets are deposited in the MetaboLights repository (ID: MTBLS13253).

### 2.3 Data analysis

Lipidomic data from host cells and TgEVs were processed and analyzed in R (v4.5.1) (R Core Team., 2023). Lipid identifiers were standardized according to accepted shorthand notation and annotated by host cell type and material (cell or TgEV). Lipidomic data were acquired in both positive and negative ionization modes using a Thermo Q Exactive Plus Orbitrap. However, only data acquired in positive ionization mode were processed and included in the analysis presented here, as most lipid classes of interest showed optimal signal and identification under these conditions.

Background signals were excluded using blank-based filtering, and retained features were required to occur in the majority of replicates within at least one host group. Low-quality samples were identified by total ion current (TIC) and principal component analysis (PCA), and one technical outlier was removed. Intensities were normalized to TIC, log_2_-transformed, and missing values imputed using a k-nearest neighbor (KNN) approach. Given the compositional nature of lipidomic data, analyses were performed on log_2_- or centered log-ratio (CLR)–transformed intensities. Global lipidome variation was assessed by PCA, and group-level differences were tested by PERMANOVA using Aitchison distance. Differential lipid abundance was assessed using moderated linear models (limma (Chion et al., 2022)) with FDR < 0.05. Fold-change thresholds (|log_2_FC| ≥ 0.58 soft, ≥1 reference) were applied for visualization. Relationships between TgEV and cellular lipidomes were quantified using Pearson correlation and paired differential analysis, with lipid class–level changes evaluated using Wilcoxon test. Lipid subclass enrichment was investigated by gene set enrichment analysis (GSEA (Subramanian et al., 2005)) based on ranked fold changes, reporting normalized enrichment scores (NES) and FDR-adjusted p-values.

Detailed descriptions of quality control, filtering criteria, imputation, compositional transformations, and statistical modelling are provided in the Supplementary Methods.

## 3 Results

### 3.1 Host cell lipidomes define distinct baseline compositions

**Figure 1:**
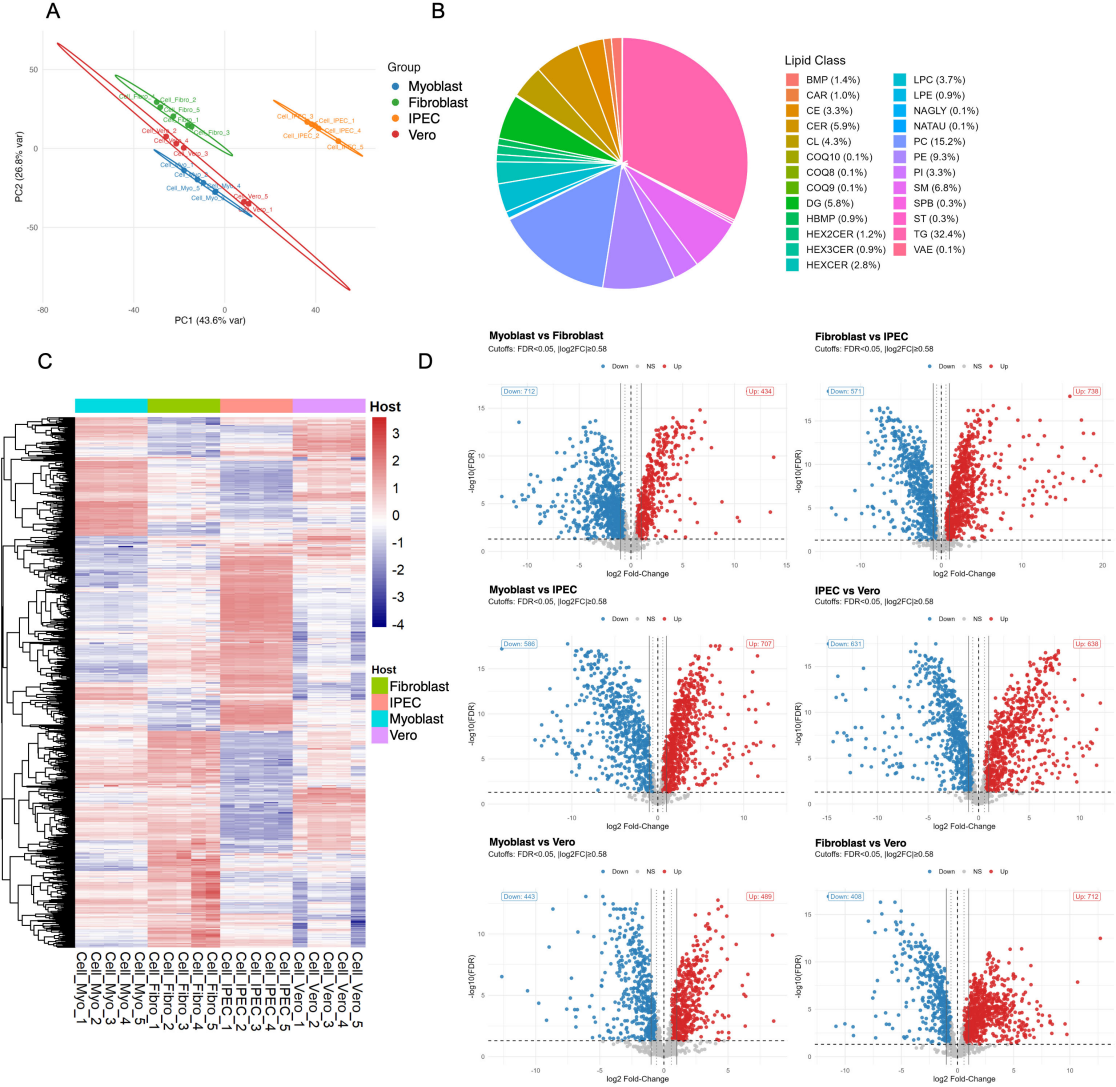
Comparative lipidomic profiling of four host cell types. **(A)** Principal component analysis (PCA) of global lipidomes from IPEC, fibroblast, myoblast, and Vero cells reveals clear clustering by cell type, indicating distinct lipid metabolic profiles. **(B)** Distribution of lipid classes across all samples, expressed as the proportion of total lipid abundance. **(C)** Heatmap hierarchically clustered by abundance. Lipid species group by host cell type, reflecting lineage-specific metabolic signatures. **(D)** Volcano plots showing differential lipid abundance in all pairwise comparisons between cell types (FDR < 0.05, |log2FC| > 1). Red and blue points indicate significantly upregulated and downregulated lipids, respectively. Extensive lipid remodelling is observed between cell types, with the largest shifts occurring between myoblasts and epithelial cells.

To establish the lipid environments in which *T. gondii* replicates, we first characterized the lipidomes of four host cell types: intestinal epithelial cells (IPEC), fibroblasts, myoblasts, and kidney epithelial cells (Vero). After processing, 1, 597 lipid species across 25 classes were quantified (**Supplementary Table S1**). Unsupervised analyses immediately revealed profound cell-type-specific patterning. Principal component analysis (PCA) showed a clear separation of samples according to their cell type of origin, indicating distinct lipidomic signatures (**Figure 1A**). The first two principal components together explained over 70% of the total variance, underscoring the robustness of these differences. This pattern was confirmed by unsupervised hierarchical clustering, where biological replicates grouped tightly to form discrete, cell type–specific clusters with minimal overlap (**Figure 1C**).

Despite this diversity, a conserved core lipidome was evident. Global lipid class distribution revealed that phosphatidylcholine (PC), triacylglycerols (TG), and phosphatidylethanolamine (PE) were the most abundant classes across all cell types (**Figure 1B**). Triacylglycerols (TG) accounted for approximately one-third of the total lipid content (32.4%), followed by phosphatidylcholine (PC, 15.2%), phosphatidylethanolamine (PE, 9.3%), sphingolipids such as sphingomyelin (SM, 6.8%) and ceramides (CER, 5.9%). Other classes like phosphatidylinositol (PI), and hexosylceramides (HexCER) contributed smaller fractions. However, beyond this shared core, we identified extensive quantitative remodelling at the species level. Differential abundance analysis revealed widespread changes, with hundreds of lipids significantly altered (FDR < 0.05) in pairwise comparisons between cell types (**Figure 1D**). The largest differences were observed in comparisons including IPEC, reflecting metabolic distinctions in this epithelial lineage.

To better understand the organization of lipid metabolism across different host cell types, we constructed reaction network maps linking individual lipid species by predicted elongation and desaturation steps. This approach revealed major differences in lipid composition and metabolic architecture among host cells (**Supplementary Figure S1**). Despite sharing a common set of major lipid classes, each host cell type displayed a unique network topology and abundance distribution. Glycerophospholipids, including PC, PE, and PI, formed large, interconnected clusters across all hosts, consistent with their central roles in membrane structure and signalling. Neutral lipids such as TG were represented as distinct, highly abundant clusters, reflecting variable storage capacities across cell types. Together, these network-level analyses demonstrate that host cells not only differ in their lipid composition but also in the organization and connectivity of their lipid metabolic pathways.

To formally assess these class-level compositional shifts, we applied centred log-ratio (CLR) transformation, which accounts for the constrained nature of lipidomic data. This analysis confirmed systematic host-specific biases (**Supplementary Table S1**; **Supplementary Figure S2**). Several lipid classes exhibited notable shifts between host cell types. Fibroblasts were consistently characterized by an accumulation of neutral and structural membrane lipids compared with IPEC cells, with higher levels of CE, CAR, LPC, and PC, while metabolites associated with sphingolipid and phospholipid metabolism (HBMP, HEXCER, and PE) were relatively reduced. A similar trend was observed when fibroblasts were compared with myoblasts or Vero cells: CE, HEXCER, CER, SM, and TG remained enriched in fibroblasts, whereas a broad range of phospholipid-related classes (HBMP, CAR, BMP, LPC, LPE, CL, DG, and PE) were comparatively lower. IPEC cells displayed a markedly distinct lipid profile compared with Vero cells, showing selective enrichment in storage and structural components such as TG and PE, but depletion across a broad spectrum of lipid classes (CE, HBMP, HEXCER, LPC, LPE, CL, DG, and PC), suggesting a more restricted lipid repertoire. Myoblasts also exhibited a distinctive metabolic signature, characterized by elevated levels of multiple lipid classes linked to energy storage and membrane dynamics (CE, HBMP, CAR, BMP, LPC, LPE, CL, DG, TG, and PC) relative to IPEC cells, yet lower levels of sphingolipid species (HEXCER, CER, and SM) and PE. Conversely, Vero cells were enriched in sphingolipids (CE, HEXCER, CER, and SM) compared with myoblasts but displayed reduced levels of several glycerophospholipid-related species (HBMP, CAR, BMP, LPC, and TG).

Collectively, these data demonstrate that host cell lipidomes are highly structured, reproducible, and cell type specific. More importantly, these host-specific compositional landscapes define the distinct lipid pools from which *T. gondii* draws resources and assembles its secreted extracellular vesicles, setting the stage for investigating host-driven differences in TgEV composition.

### 3.2 TgEV characterization

**Figure 2:**
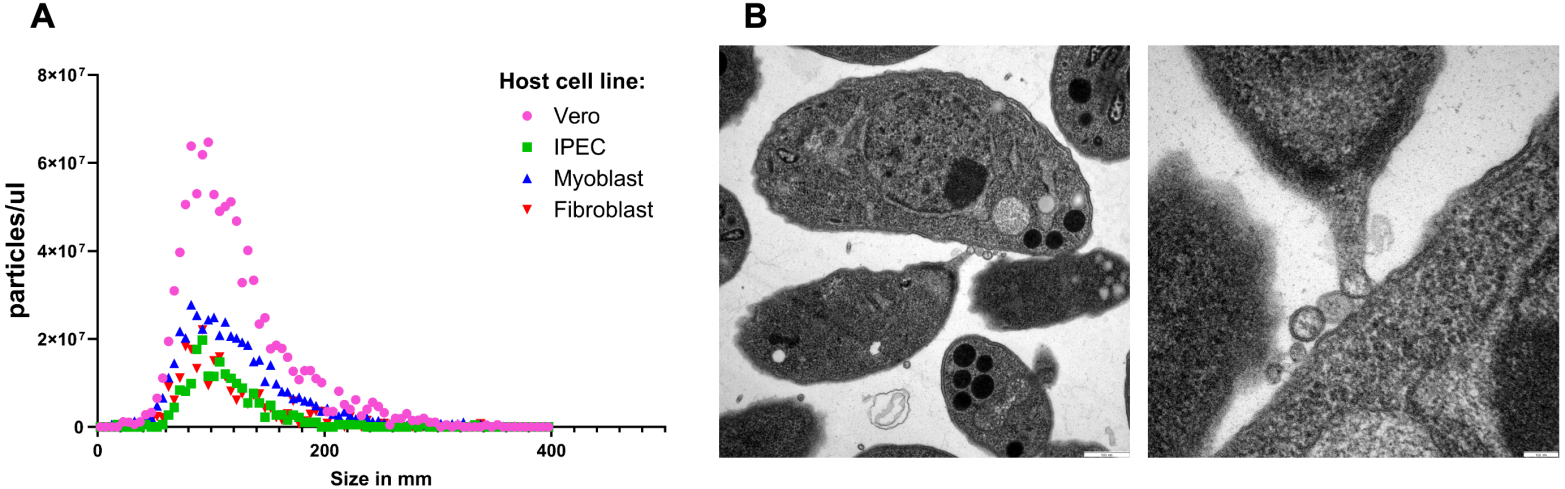
Characterization of TgEVs. **(A)** Nanoparticle tracking analysis of EVs isolated from cultures of *T. gondii*–infected IPEC, fibroblast, myoblast, and Vero cells. Size distribution profiles show heterogeneous vesicle populations predominantly ranging from 50–200 nm in diameter, consistent with small EVs. **(B)** Transmission electron microscopy images of TgEV from fibroblast preparations confirm the presence of round, lipid bilayer–enclosed vesicles with characteristic morphology (scale bars: 100 nm).

To investigate the lipid composition of TgEVs, we first confirmed them across infections in the four host cell types: IPEC, fibroblast, myoblast, and Vero cells. Nanoparticle tracking analysis revealed a heterogeneous size distribution in all samples, with particle diameters primarily ranging from 50–200 nm, consistent with small EVs (**Figure 2A**). Vesicle concentration differed significantly across host cell types, with Vero cells producing the highest number of particles per unit volume, followed by myoblasts, fibroblasts, and IPEC cells. Transmission electron microscopy confirmed the presence of vesicles with typical cup-shaped morphology, often budding from the host cell membrane or observed in proximity to *T. gondii* tachyzoites (**Figure 2B**), validating the isolation of bona fide TgEV populations.

### 3.3 Global lipid class distribution in *T. gondii* EVs

To determine whether the TgEV lipid composition is a passive reflection of the host environment or a result of selective, parasite-directed packaging, we profiled TgEVs released from tachyzoites grown in the host cell types. Following outlier removal (TgEV_Myo_2) and data processing, 194 lipid species across 15 classes were quantitatively analysed (**Supplementary Table S2**).

**Figure 3:**
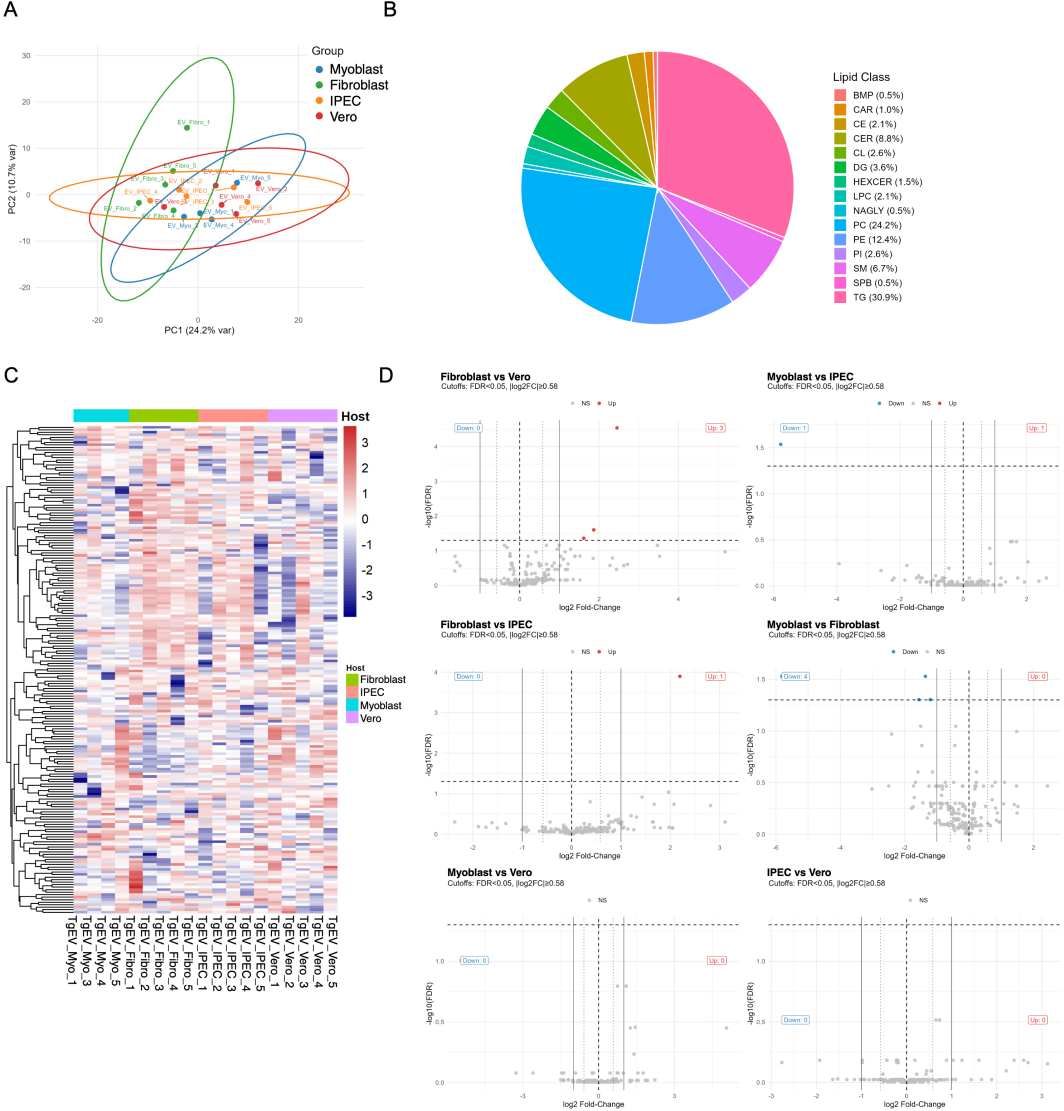
Lipidomic profiling reveals host cell–specific signatures in TgEVs. **(A)** Principal component analysis (PCA) of the TgEVs lipidomes from four host cell types demonstrates clear segregation by cell origin. **(B)** Global lipid class distribution across all TgEVs samples, expressed as a percentage of total lipid abundance. **(C)** Heatmap hierarchically clustered by abundance. **(D)** Volcano plots of pairwise comparisons reveal significantly altered lipid species (FDR < 0.05, |log2FC| > 1) between TgEVs samples derived from different host cell types.

Notably, the global TgEV lipidome is largely conserved across all host conditions. Principal component analysis revealed substantial overlap among TgEVs from the four different host cell types, with no clear separation by host of origin (**Figure 3A**). This core conservation was reflected in the lipid class distribution, which was remarkably consistent across all TgEV samples (**Figure 3B**). PC and PE were the dominant classes, collectively accounting for more than 35% of the total lipid abundance, followed by TG, SM, and CER. Despite this overarching conservation, unsupervised hierarchical clustering heatmap revealed subtle signatures. TgEV lipid profiles from each host type formed distinct, tight clusters (**Figure 3C**), indicating that the packaging machinery produces a highly reproducible lipid profile, albeit with minor variations influenced by the host environment. However, these clusters showed considerable similarity and did not indicate strict host partitioning. The limited nature of these host-dependent differences was confirmed at the species level (**Figure 3D**). Differential abundance analysis identified only a sparse subset of lipid species that were significantly altered in pairwise host comparisons (FDR < 0.05, **Figure 3D**). The effect sizes for these changes were generally modest, reinforcing that the TgEV lipidome is composed of a strongly conserved core, with only a handful of species exhibiting host-influenced abundance.

To explore the metabolic organization of TgEV lipidomes, we reconstructed lipid reaction networks integrating chain length, saturation, and known biosynthetic relationships (**Supplementary Figure S3**). In contrast to the dense and interconnected networks observed in host cell lipidomes (**Supplementary Figure S1**), EV lipid networks were markedly sparser and exhibited lower overall connectivity, indicating a reduced metabolic diversity in vesicular lipid cargo. While glycerophospholipids (PC, PE, PI) and neutral lipids (TG) formed conserved network structures, their abundance and connectivity varied markedly. Despite these network-level nuances, a class-level compositional analysis confirms the robustness of the core TgEVs lipidome. When we directly compared class abundances between hosts using CLR-transformed data (**Supplementary Table S1**; **Supplementary Figure S4**), the overall rate of conservation was very high. The vast majority of lipid classes showed non-significant, near-zero changes across all host-host contrasts. The single, reproducible exception was triacylglycerol (TG), which exhibited modest but significant host-dependent tuning (median |log_2_FC| ≈ 0.2–0.6). Collectively, our results indicate that TgEVs possess a conserved lipid core, dominated by PC, PE, and sphingolipids, that is maintained across all host environments. Host-specific effects are limited, mainly fine-tuning the abundance of storage lipids such as TG.

### 3.4 Multivariate analysis

**Figure 4:**
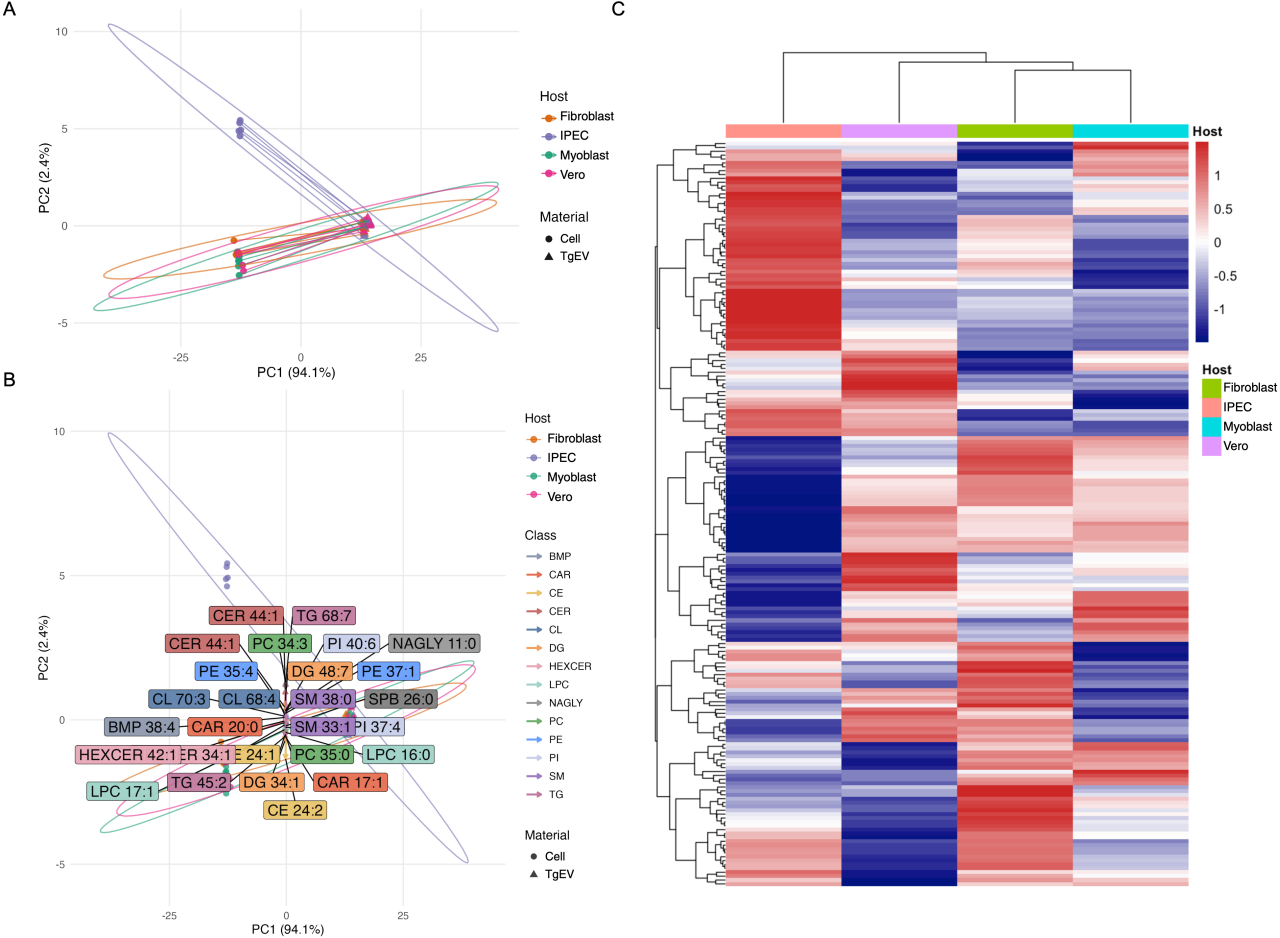
Principal component and clustering analyses reveal distinct lipidomic profiles of TgEVs compared to host cell lipidomes. **(A)** Principal component analysis (PCA) of lipidomes from host cells (circles) and TgEVs (EVs, triangles) derived from fibroblast, IPEC, myoblast, and Vero cell cultures. **(B)** PCA loadings plot highlighting lipid species that contribute most strongly to the separation between TgEVs and cellular lipidomes. **(C)** Hierarchical clustering heatmap of lipid species abundances across TgEVs and cell samples.

Having established that the TgEV lipidome is largely host-independent, we next asked if it simply mirrors the lipid composition of its host cell. To test this directly, we performed a comparative lipidomic analysis of TgEVs and their corresponding host cells across all four host types. This analysis revealed a substantial overlap between the two compartments, with 192 lipid species in common, which were subsequently used for comparative analyses (**Supplementary Table S3**). Principal component analysis of the combined dataset yielded a decisive result: while TgEV and cell samples from the same host clustered in proximity, they were consistently and clearly separated along the first principal component, which accounted for over 90% of the total variance (**Figure 4A**). This profound separation demonstrates that the TgEV lipidome is not a diluted sample of the host cell but constitutes a distinct lipid subset. Lipid species driving the separation included a mixture of glycerophospholipids (PC, PE, PI), sphingolipids (SM, CER, HexCER), and neutral lipids (TG, DG) (**Figure 4B**), indicating that the selective packaging mechanism acts across multiple lipid categories rather than targeting a single class.

Hierarchical clustering of lipid abundances confirmed that TgEVs and cellular lipidomes formed discrete clusters within each host type (**Figure 4C**), highlighting selective lipid sorting processes during TgEVs biogenesis.

The profound separation in the PCA demonstrated that the TgEVs lipidome constitutes a distinct lipid subset. To further quantify this relationship, we assessed the correlation of shared lipid species between each TgEV–host cell pair. Across all host types, correlation coefficients were consistently low (Pearson r: 0.21–0.34), with coefficients of determination (R² = 0.06–0.12) confirming that host cell abundance is a poor predictor of its level in TgEVs (**Supplementary Figure S5A–D**).

**Figure 5:**
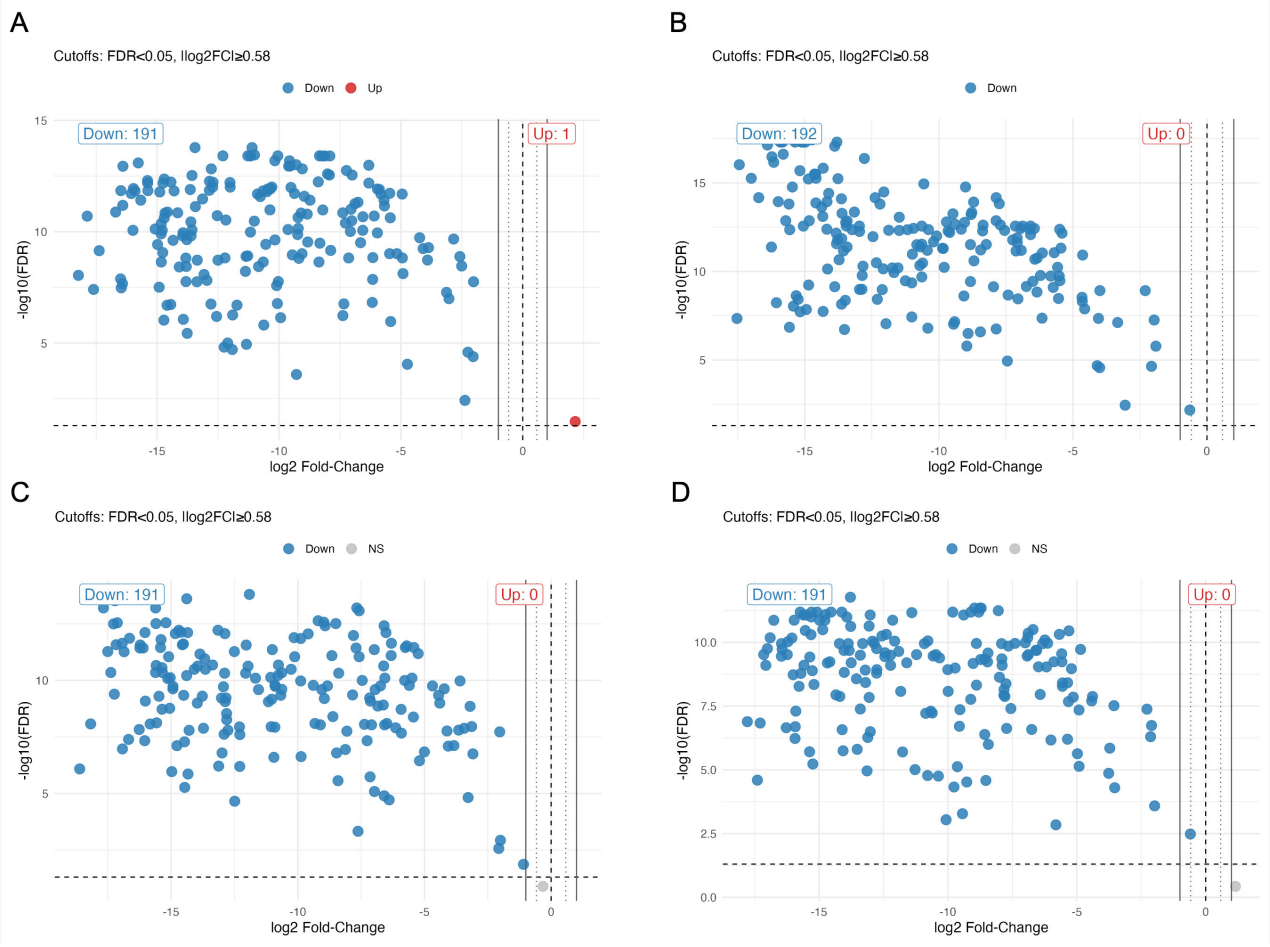
Differential lipid abundance between TgEVs and host cells. Volcano plots showing pairwise comparisons of lipid species between TgEVs and their corresponding host cells for each host cell type: **(A)** IPEC, **(B)** fibroblast, **(C)** Vero, and **(D)** myoblast. Each point represents an individual lipid species, plotted according to its log_2_ fold change (EV vs. cell) and –log_10_(FDR). Horizontal and vertical dashed lines indicate significance thresholds (FDR < 0.05 and |log_2_FC| ≥ 0.58, respectively).

To directly compare lipid abundance between TgEVs and their corresponding host cells, we performed differential abundance analysis for all detected lipid species (**Figure 5**). Across all cell types, volcano plots revealed a consistent pattern in which the vast majority of lipids were significantly less abundant in TgEVs. Specifically, between 191 and 192 lipid species showed significant depletion (FDR < 0.05, |log_2_FC| ≥ 0.58) in TgEVs relative to the cellular fraction, while none or only a single species was significantly enriched. This pronounced asymmetry indicates that the lipid composition of TgEVs differs substantially from that of the producing host cells, with most shared lipid species present at lower relative levels in the vesicles.

**Figure 6:**
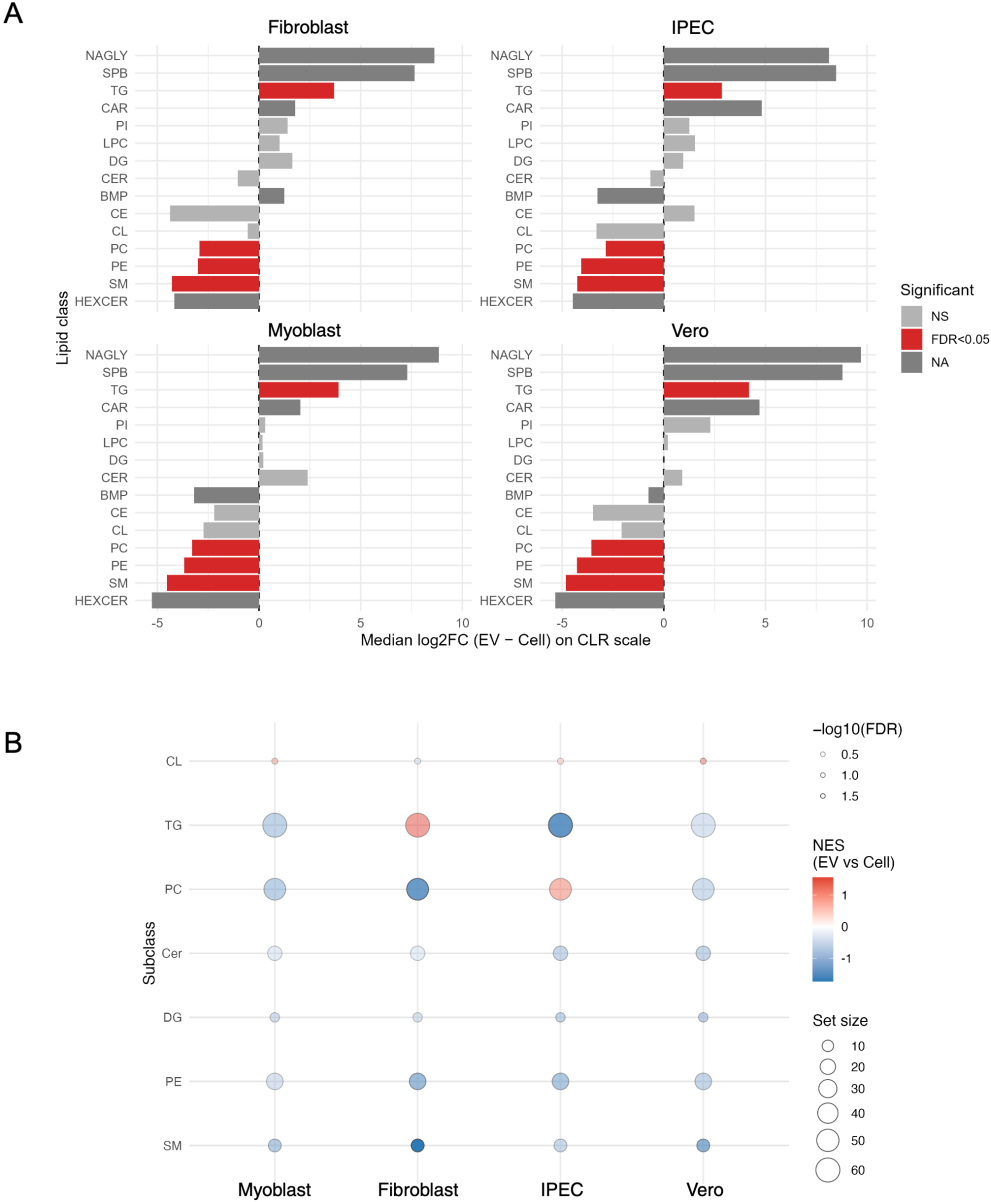
Selective enrichment and depletion of lipid classes in TgEVs relative to host cells. **(A)** Compositional log-ratio (CLR) analysis of lipid class abundances comparing TgEVs and their parental cells for each host cell type. Bars indicate median log2 fold changes (TgEVs versus cell), with red bars representing significant differences (FDR < 0.05), grey bars non-significant changes (NS), and dark grey indicating lipid classes not testable (NA). **(B)** Lipid set enrichment analysis of major lipid subclasses comparing TgEVs and cell lipidomes. Bubble size represents the number of species per subclass, and colour indicates normalized enrichment score (NES), with red denoting enrichment in EVs and blue depletion.

To definitively identify the rules governing this selectivity, we performed a compositional log-ratio (CLR) analysis. This revealed a clear and consistent signature of lipid enrichment and depletion (**Figure 6A**). Key structural and signalling lipids, including PC, PE and SM, were significantly enriched in cells across all host types. Conversely, lipids primarily associated with energy storage, such as TG, were systematically enriched in TgEvs. While this core enrichment pattern was universal, we observed host-dependent modulation of its magnitude. Finally, a set enrichment analysis at the subclass level consolidated these findings, revealing a highly consistent pattern across all EV samples (**Figure 6B**). For instance, TG enrichment was more pronounced in TgEVs from fibroblast, whereas PC were more strongly enriched in TgEVs from IPEC.

## 4 Discussion

Parasitic survival is often described as a battle for resources between parasite and host (Woolhouse et al., 2002), but in the case of *T. gondii*, it is more accurate to think of it as a negotiation between the two, a metabolic dialogue between parasite and host that plays out at the level of membranes and intercellular signals. Our lipidomic analysis of both host cells and *T. gondii*-derived extracellular vesicles reveals that this dialogue is far more intricate than a unidirectional acquisition of nutrients by the parasite. Instead, it reflects a highly regulated process in which the lipid environments of host cells provide substrates that *T. gondii* can access through host lipid scavenging and parasite *de novo* synthesis (Coppens et al., 2000; Coppens and Botté, 2020; Shunmugam et al., 2022). However, these substrates do not fully determine the final vesicle lipidome. The parasite, in turn, selectively repackages and redistributes these lipids to serve its own good, including the manipulation of its surroundings in its favour. This study builds upon our previous characterization of the proteomic landscape of TgEVs, which revealed a conserved core of parasite-derived effectors and host-interacting proteins across diverse host cell types (Cruz-Bustos et al., 2025). Together, these complementary datasets provide a multidimensional view of TgEVs biology, highlighting that selective cargo packaging extends beyond proteins to the lipid constituents that define vesicle identity and function.

### 4.1 A metabolically diverse, host specific landscape

Our lipidomic profiling reveals that the host cell is far from a passive lipid background for *T. gondii* infection; rather, it presents a diverse metabolic landscape that likely dictates parasitic strategy. These findings extend recent evidence that host lipid metabolism is a key determinant of parasite replication dynamics and developmental plasticity (Walsh et al., 2022; He et al., 2024; Amiar et al., 2020) and they provide a mechanistic framework for understanding how *T. gondii* senses and responds to metabolic cues within its intracellular niche.

The marked differences we observed among cell types suggest that the parasite encounters fundamentally distinct lipid landscapes that could differentially influence its biology. Myoblasts and fibroblasts emerged as notably lipid-rich environments. Myoblasts were characterized by elevated levels of a broad spectrum of lipids, including energy storage molecules (CE, TG), membrane phospholipids (PC, LPC, LPE), and intermediates of lipid metabolism and signalling (CAR, BMP, HBMP). Fibroblasts also accumulated neutral lipids (CE, TG) and specific structural and signalling lipids like HEXCER, CER, and SM. In contrast, Vero cells displayed a more specialized profile, enriched in sphingolipids (HEXCER, CER, SM) but lower in key glycerophospholipids and triacylglycerol. IPEC cells presented the most restricted lipid repertoire, showing depletion across many lipid classes compared to the others. Although the lipid profiles clearly distinguish between host cell types, they should not be regarded as unique molecular fingerprints of cell identity. Instead, they reflect lineage-specific metabolic organization: myoblasts and fibroblasts display broad, lipid-rich profiles dominated by neutral lipids and diverse glycerophospholipids, whereas epithelial cells show more specialized lipidomes, Vero cells being enriched in sphingolipids and IPEC cells exhibiting a reduced overall lipid diversity. This heterogeneity has direct consequences for *T. gondii*, known to both synthesize lipids *de novo* and scavenge them from the host (Coppens and Botté, 2020; Shunmugam et al., 2022). The lipid-rich environments of myoblasts and fibroblasts, replete with neutral lipids and diverse phospholipids, are consistent with a more scavenging-heavy metabolic strategy, which has been associated with rapid replication and expansion of the parasitophorous vacuole. Conversely, the distinct sphingolipid specialization of Vero cells and the restricted lipid repertoire of IPEC cells may force a significant metabolic shift. As described in the literature (Shunmugam et al., 2022; He et al., 2024), in these contexts, the parasite likely increases scavenging and *de novo* synthesis to overcome lipid scarcity or imbalance, an energetically costly adaptation that could curtail replication rates. We propose that this fundamental trade-off between scavenging and synthesis, dictated by host cell lipid composition, serves as a key regulator of parasite resource allocation, in line with previous observations that host cell context drives tissue-specific growth rates and stage conversion (He et al., 2024). This, in turn, may directly influence critical downstream phenotypes such as replication speed, vacuole morphology, and ultimately, the decision between acute proliferation and chronic persistence, explaining the noted tissue-specific outcomes of toxoplasmosis. Taken together, our dataset shows that host cell lipidomes differ markedly across lineages, implying that intracellular tachyzoites experience distinct lipid availability and membrane precursor pools. Such differences align with prior work showing that host lipid uptake, lipid droplet mobilization, and parasite lipid biosynthetic plasticity shape replication efficiency and stage conversion. In this context, our observation that TgEV lipidomes remain comparatively conserved across host backgrounds suggests that EV lipid export is an actively regulated output downstream of host metabolic variation, rather than a passive readout of host membrane composition.

### 4.2 A conserved TgEVs lipidome reveals active parasite curation

If host cells set the stage, TgEVs are the parasite’s reply, and our findings indicate that this reply is anything but passive. Despite being produced within host-defined metabolic environments, TgEVs consistently diverged from their source cells in lipid composition. Principal component analysis showed robust separation between cellular and TgEVs lipidomes, and shared lipid species correlated only weakly, indicating that vesicle composition is not simply a snapshot of the host membrane but the product of selective sorting. As TgEVs were harvested during host cell-free incubation following parasite egress, the source of the immediate membrane for vesicle biogenesis is most likely parasite-derived. However, parasite membranes themselves are shaped by a composite lipid economy that incorporates both *de novo* synthesis by the parasite and scavenging of host lipids. Therefore, TgEV lipids are best interpreted as a parasite-assembled output with potentially mixed metabolic provenance. Importantly, the weak TgEV–cell correlations, together with the strong multivariate separation, support the conclusion that the composition of TgEVs reflects active parasite curation rather than passive copying of host membranes.

A limitation of our study is that TgEVs were harvested after parasite egress and during a defined host cell–free incubation, which was chosen to minimize co-isolation of host-derived vesicles. Accordingly, the lipidomic conclusions presented here specifically describe post-egress, extracellular tachyzoite-derived EVs and do not directly resolve the lipid composition of EVs generated within infected cells, such as those associated with the parasitophorous vacuole or intravacuolar network. Addressing intracellular TgEV lipid composition will require future approaches combining parasite-specific EV immuno-capture with stable isotope tracing to partition host-derived and parasite-synthesized lipid contributions.

A closer inspection of the TgEVs lipid composition reveals a remarkably non-random composition. TgEVs are dominated by glycerophospholipids, particularly PC and PE, which together account for more than one third of the vesicle lipidome. These molecules are essential for maintaining vesicle membrane structure, curvature, and flexibility, all of which are critical for efficient budding, cargo encapsulation, and vesicle target cell interactions (Yáñez-Mó et al., 2015; Skotland et al., 2020). The presence of sphingolipids, including sphingomyelin (SM, CER and HEXCER) further points to the formation of lipid rafts, specialized microdomains that regulate cargo sorting and vesicle fusion dynamics (van Meer et al., 2008; Hallal et al., 2022). Importantly, TG constitute nearly one third of the TgEV lipidome, a relatively high proportion compared to most mammalian EVs, suggesting a targeted enrichment of neutral lipids. This may serve multiple functions, such as stabilizing vesicle membranes, altering vesicle density and flexibility, or supplying an energy reservoir for recipient cells, a feature that could be particularly relevant in the metabolically constrained intracellular niche of *T. gondii*, once such vesicles interact with infected or neighbouring cells.

Although host cells provide the lipid building blocks, *T. gondii* appears to exert tight control over TgEV lipid biogenesis, producing vesicles with a conserved lipid profile across distinct host environments. Hierarchical clustering and pairwise differential abundance analyses reveal minimal host-specific variation, with only a handful of lipid classes showing significant shifts. The most consistent difference was the enrichment of TG, highlighting a recurrent sorting preference independent of the host lipid milieu. The conservation of TgEV lipid composition suggests that *T. gondii* prioritizes a specific vesicle identity, one optimized for stability, signalling, and host manipulation, rather than passively reflecting the metabolic background (Skotland et al., 2020; Haraszti et al., 2016; van Niel et al., 2022). Such selective lipid control is likely to serve several adaptive purposes. By regulating the lipid composition of EVs, *T. gondii* may enhance vesicle resilience in the extracellular milieu, optimize membrane fusion with target cells, and fine-tune the delivery of effector molecules (Llorente et al., 2013; van Niel et al., 2022). Moreover, lipid signatures themselves may play signalling roles: exposed phosphatidylserine, for instance, can promote uptake by phagocytic cells while dampening inflammatory responses and ceramide-enriched domains can facilitate the packaging of virulence-associated proteins (Skotland et al., 2025). The enrichment of neutral lipids such as TG further suggests a potential role in metabolic modulation, possibly priming them for infection or altering their nutrient availability to support parasite growth.

One might expect that the host environment would imprint strongly on TgEV composition, yet our results show the opposite. At the lipid class level, TgEV profiles were highly conserved across host cell types, with only subtle shifts, most notably in TG abundance, distinguishing them. This suggests that *T. gondii* produces EVs with a conserved lipid profile regardless of the metabolic background. Such conservation likely reflects the evolutionary importance of EV-mediated communication for the parasite’s life cycle: in order to reliably manipulate host immunity and signalling, TgEVs must maintain a consistent molecular architecture.

### 4.3 Towards a fresh look at parasite-host communication

Together, our findings redefine *T. gondii* not merely as an adaptor to its metabolic environment, but as an active architect that reshapes it through lipid-based communication. Host cell lipid composition provides the raw material, but the parasite repurposes that material into EVs with tightly controlled, functionally tuned lipidomes. This selective lipid remodelling may serve multiple purposes: enhancing vesicle stability, promoting targeted delivery, or modulating host immune pathways to create a more permissive niche. It also underscores the power of lipidomics to reveal a regulatory layer of host–parasite interaction that is complementary to, yet distinct from, proteomic or transcriptomic views.

This lipid-centric view directly complements our previous proteomic characterization of *T. gondii* EVs, which revealed a highly conserved core of proteins across vesicles derived from diverse host cell types (Cruz-Bustos et al., 2025). That earlier work demonstrated that TgEVs are not passive carriers, but vehicles deliberately loaded with invasion-associated proteins and factors that reprogram host translation, stress response, and metabolic pathways. The present lipidomic data reveal that this selective control extends beyond proteins to the very fabric of the vesicle membrane itself. By integrating these two layers of evidence, a model emerges: the parasite not only customizes the molecular messages encoded in the vesicle cargo but also engineers the lipid scaffolding that determines vesicle stability, trafficking, and cellular uptake.

Looking forward, several key questions emerge. What molecular machinery enables and promotes lipid selection during TgEV biogenesis? How do specific lipid species contribute to host signalling modulation? And can interference with lipid sorting disrupt parasite communication and limit infection? Addressing these questions will require integrative approaches that combine lipidomics with functional assays, genetic manipulation, and host response profiling.

## 5 Conclusions

This study provides the first depth lipidomic characterization of *T. gondii* extracellular vesicles and establishes how their lipid composition relates to both parasite biology and the metabolic context of the host cell. By systematically comparing host cell lipidomes, TgEVs lipid composition, and the relationship between the two across diverse cellular environments, our findings reveal three key principles: (i) host cells exhibit distinct lipidomic signatures that define the metabolic landscape available to *T. gondii*; (ii) TgEVs possess a conserved yet selectively curated lipid profile enriched in structural and bioactive lipids; and (iii) TgEV lipid composition is shaped by selective sorting processes that highlight functional specialization. As this study demonstrates, the lipid dimension of parasite biology is not merely structural; it is communicative, adaptive, and deeply intertwined with the evolutionary success of *T. gondii*.

## Data Availability

The datasets presented in this study can be found in online repositories. The names of the repository/repositories and accession number(s) can be found below: https://www.ebi.ac.uk/metabolights/, MTBLS13253.

## Supplementary material

Supplementary Figure 1 Global lipid reaction network topology across four host cell types. (A–D) Lipid reaction networks of the most abundant lipid species in **(A)** IPEC, **(B)** fibroblast, **(C)** myoblast, and **(D)** Vero cells, reconstructed based on known biosynthetic and degradation pathways. Nodes represent individual lipid species, scaled by carbon chain length and colored by lipid class. Node size indicates relative abundance (z-score normalized), while edges represent enzymatic transformations. Distinct network architectures and dominant lipid clusters highlight cell type–specific lipid metabolic organization.

Supplementary Figure 2 Class-level differential lipid abundance between host cell types. Compositional log-ratio (CLR) analysis of lipid class abundance for all pairwise comparisons between host cell types. Bars indicate median log2 fold changes in lipid class abundance (host A – host B). Red bars denote statistically significant differences (FDR < 0.05), grey bars represent non-significant changes (NS), and dark grey indicates classes not testable, not enough lipid species for a statistical test (NA).

Supplementary Figure 3 Metabolic network topology of TgEV lipidomes. (A–D) Network reconstruction of the most abundant EV lipid species from T. gondii–infected (A) IPEC, (B) fibroblast, (C) Vero, and (D) myoblast. Nodes represent individual lipid species, scaled by chain length and coloured by lipid class. Node size reflects relative abundance (z-score normalized), while edges indicate known enzymatic elongation or desaturation reactions.

Supplementary Figure 4 Comparative lipid class abundance across TgEVs. Compositional log-ratio (CLR) analysis of lipid class abundances in pairwise comparisons of EV samples from different host cell types. Bars represent median log2 fold changes (host A – host B) for each lipid class. Red bars indicate statistically significant differences (FDR < 0.05), grey bars denote non-significant changes (NS), and dark grey indicates classes not testable (NA).

Supplementary Figure 5 Correlation between host cell and T. gondii EV lipidomes reveals moderate overlap and selective lipid sorting. Scatter plots show the correlation of shared lipid species between EVs and host cells for each cell type: (A) IPEC, (B) fibroblast, (C) Vero, and (D) myoblast. Each dot represents the mean log2 abundance of a shared lipid species in the EV (y-axis) versus the cell (x-axis).

Supplementary Table 1 Lipidomic dataset and statistical analysis for host cell samples. This table contains quantitative lipidomic data and statistical analyses for host cell samples infected with *Toxoplasma gondii*. The Raw sheet includes unprocessed ion intensity values. The Processed_Cell_Log2_Imp sheet provides the log_2_-transformed and imputed lipid abundance matrix after data filtering. The Summary_Pairwise and subsequent sheets summarize pairwise comparisons between host cell types performed using the limma framework on CLR-transformed data. The Cell_Class_Pairs_CLR sheet reports lipid class–level median log_2_ fold changes and Wilcoxon test statistics, indicating class-specific differences between host cell types.

Supplementary Table 2 Lipidomic dataset and statistical analysis for TgEVs. This table summarizes the quantitative lipidomic data and host-dependent comparisons for *T. gondii* extracellular vesicles. The Raw sheet lists unprocessed EV lipid intensities and Processed_EV_Log2_Imp (2) presents the log_2_-transformed and imputed dataset after normalization and quality control. The Summary sheet provides an overview of differential abundance results across host cell origins. Pairwise comparison sheets show log_2_ fold changes and FDR-adjusted *p*-values obtained from CLR-transformed data. The EV_Class_Pairs_CLR sheet reports class-level enrichment analyses, including median log_2_ fold changes and significance levels for each lipid class across host contrasts.

Supplementary Table 3 Integrated and paired analyses of host cell and TgEV lipidomes. This table integrates lipidomic datasets from host cells and *T. gondii*–derived EVs to assess lipid selection, conservation, and host-specific enrichment. The Raw sheet lists unprocessed values, while Intersection_EV_Log2_Imp and Intersection_Cell_Log2_Imp contain the log_2_-transformed and imputed abundance matrices for lipids detected in both EVs and host cells. The Paired_Myo, Paired_Fibro, Paired_IPEC, and Paired_Vero sheets report paired analyses comparing EVs with their respective host cells. The Class_by_Host_CLR sheet summarizes median class-level log_2_ fold changes (EV – Cell) with FDR-adjusted Wilcoxon *p*-values per host. GSEA_EV_vs_Cell presents pathway- or class-level enrichment analyses, while PerHost_Scatter_Summary and Delta_EVminusCell summarize per-host lipid correlations and abundance differences. The Class_Summary sheet compiles the overall lipid class statistics across all hosts.

## References

[B1] Amiar S. Katris N. J. Berry L. Dass S. Duley S. Arnold C.-S. . (2020). Division and adaptation to host environment of apicomplexan parasites depend on apicoplast lipid metabolic plasticity and host organelle remodeling. Cell Rep. 30, 3778–3792.e9. doi: 10.1016/j.celrep.2020.02.072, PMID: 32187549

[B2] Brent A. Shirmast P. McMillan N. A. J. (2025). Extracellular vesicle lipids and their role in delivery. J. Extracell Biol. 4, e70064. doi: 10.1002/jex2.70064, PMID: 40552106 PMC12183401

[B3] Chion M. Carapito C. Bertrand F. (2022). Towards a more accurate differential analysis of multiple imputed proteomics data with Mi4limma. Methods Mol. Biol. 2426, 131–140. doi: 10.1007/978-1-0716-1967-4_7, PMID: 36308688

[B4] Colombo M. Raposo G. Théry C. (2014). Biogenesis, secretion, and intercellular interactions of exosomes and other extracellular vesicles. Annu. Rev. Cell Dev. Biol. 30, 255–289. doi: 10.1146/annurev-cellbio-101512-122326, PMID: 25288114

[B5] Coppens I. Botté C. (2020). “ Biochemistry and metabolism of *Toxoplasma gondii*: Lipid synthesis and uptake,” in Toxoplasma gondii, 3rd ed. Eds. Weiss L. M. Kim K. B. ( Academic Press (Elsevier)), 367–395. doi: 10.1016/B978-0-12-815041-2.00008-6

[B6] Coppens I. Sinai A. P. Joiner K. A. (2000). *Toxoplasma gondii* exploits host low-density lipoprotein receptor-mediated endocytosis for cholesterol acquisition. J. Cell Biol. 149, 167–180. doi: 10.1083/jcb.149.1.167, PMID: 10747095 PMC2175092

[B7] Core Team R. (2023). R: A language and environment for statistical computing (Vienna, Austria: R Foundation for Statistical Computing). Available online at: https://www.r-project.org/ (Accessed January 26, 2026).

[B8] Cruz-Bustos T. Feix A. S. Hummel K. Schlosser S. Razzazi-Fazeli E. Joachim A. (2025). The proteomic landscape of *Toxoplasma gondii* extracellular vesicles across diverse host cell types. Front. Cell. Infect Microbiol. 15. doi: 10.3389/fcimb.2025.1565684, PMID: 40171158 PMC11958994

[B9] Domingues N. Pires J. Milosevic I. Raimundo N. (2025). Role of lipids in interorganelle communication. Trends Cell Biol. 35, 46–58. doi: 10.1016/j.tcb.2024.04.008, PMID: 38866684 PMC11632148

[B10] Drotleff B. Lämmerhofer M. (2019). Guidelines for selection of internal standard-based normalization strategies in untargeted lipidomic profiling by LC-HR-MS/MS. Analytic Chem. 91, 9836–9843. doi: 10.1021/acs.analchem.9b01505, PMID: 31241926

[B11] Drotleff B. Roth S. R. Henkel K. Calderón C. Schlotterbeck J. Neukamm M. A. . (2020). Lipidomic profiling of non-mineralized dental plaque and biofilm by untargeted UHPLC-QTOF-MS/MS and SWATH acquisition. Analytic Bioanalytic Chem. 412, 2303–2314. doi: 10.1007/s00216-019-02364-2, PMID: 31942654 PMC7118048

[B12] Dubey J. P. (2008). The history of *Toxoplasma gondii*—The first 100 years. J. Eukaryotic Microbiol. 55, 467–475. doi: 10.1111/j.1550-7408.2008.00345.x, PMID: 19120791

[B13] Dubey J. P. (2023). *Toxoplasmosis of animals and humans* (3rd ed.) (Boca Raton: CRC Press). doi: 10.1201/9781003199373

[B14] Fernandez-Becerra C. Xander P. Alfandari D. Dong G. Aparici-Herraiz I. Rosenhek-Goldian I. . (2023). Guidelines for the purification and characterization of extracellular vesicles of parasites. J. Extracell Biol. 2, e117. doi: 10.1002/jex2.117, PMID: 38939734 PMC11080789

[B15] Fyfe J. Casari I. Manfredi M. Falasca M. (2023). Role of lipid signalling in extracellular vesicle–mediated cell-to-cell communication. Cytokine Growth Factor Rev. 73, 20–26. doi: 10.1016/j.cytogfr.2023.08.006, PMID: 37648617

[B16] Ghadami S. Dellinger K. (2023). The lipid composition of extracellular vesicles: Applications in diagnostics and therapeutic delivery. Front. Mol. Biosci. 10. doi: 10.3389/fmolb.2023.1198044, PMID: 37520326 PMC10381967

[B17] Haase S. Temesvari L. Zhang K. (2023). Editorial: Lipids in host and protozoan parasite interaction. Front. Cell. Infect Microbiol. 13. doi: 10.3389/fcimb.2023.1334002, PMID: 38045759 PMC10693326

[B18] Hallal S. Tűzesi Á. Grau G. E. Buckland M. E. Alexander K. L. (2022). Understanding the extracellular vesicle surface for clinical molecular biology. J. Extracell Vesicles 11, e12260. doi: 10.1002/jev2.12260, PMID: 36239734 PMC9563386

[B19] Hannun Y. A. Obeid L. M. (2008). Principles of bioactive lipid signalling: Lessons from sphingolipids. Nat. Rev. Mol. Cell Biol. 9, 139–150. doi: 10.1038/nrm2329, PMID: 18216770

[B20] Haraszti R. A. Didiot M. C. Sapp E. Leszyk J. Shaffer S. A. Rockwell H. E. . (2016). High-resolution proteomic and lipidomic analysis of exosomes and microvesicles from different cell sources. J. Extracell Vesicles 5, 32570. doi: 10.3402/jev.v5.32570, PMID: 27863537 PMC5116062

[B21] Harayama T. Riezman H. (2018). Understanding the diversity of membrane lipid composition. Nat. Rev. Mol. Cell Biol. 19, 281–296. doi: 10.1038/nrm.2017.138, PMID: 29410529

[B22] He T.-Y. Li Y.-T. Liu Z.-D. Cheng H. Bao Y.-F. Zhang J.-L. (2024). Lipid metabolism: The potential targets for toxoplasmosis treatment. Parasites Vectors 17, 111. doi: 10.1186/s13071-024-06213-9, PMID: 38448975 PMC10916224

[B23] Joiner K. A. Roos D. S. (2002). Secretory traffic in the eukaryotic parasite *Toxoplasma gondii*: Less is more. J. Cell Biol. 157, 557–563. doi: 10.1083/jcb.200112144, PMID: 12011107 PMC2173860

[B24] Laliberté J. Carruthers V. B. (2008). Host cell manipulation by the human pathogen *Toxoplasma gondii*. Cell. Mol. Life Sci. 65, 1900–1915. doi: 10.1007/s00018-008-7556-x, PMID: 18327664 PMC2662853

[B25] Llorente A. Skotland T. Sylvänne T. Kauhanen D. Róg T. Orłowski A. . (2013). Molecular lipidomics of exosomes released by PC-3 prostate cancer cells. Biochim. Biophys. Acta (BBA) – Mol. Cell Biol. Lipids 1831, 1302–1309. doi: 10.1016/j.bbalip.2013.04.011, PMID: 24046871

[B26] Muro E. Atilla-Gokcumen G. E. Eggert U. S. (2014). Lipids in cell biology: How can we understand them better? Mol. Biol. Cell 25, 1819–1823. doi: 10.1091/mbc.e13-09-0516, PMID: 24925915 PMC4055261

[B27] Quiarim T. M. Maia M. M. da Cruz A. B. Taniwaki N. N. Namiyama G. M. Pereira-Chioccola V. L. (2021). Characterization of extracellular vesicles isolated from types I, II and III strains of *Toxoplasma gondii*. Acta Tropica 219, 105915. doi: 10.1016/j.actatropica.2021.105915, PMID: 33861971

[B28] Ramírez-Flores C. J. Cruz-Mirón R. Mondragón-Castelán M. E. González-Pozos S. Ríos-Castro E. Mondragón-Flores R. (2019). Proteomic and structural characterization of self-assembled vesicles from excretion/secretion products of *Toxoplasma gondii*. J. Proteomics 208, 103490. doi: 10.1016/j.jprot.2019.103490, PMID: 31434009

[B29] Shunmugam S. Arnold C.-S. Dass S. Katris N. J. Botté C. Y. (2022). The flexibility of Apicomplexa parasites in lipid metabolism. PloS Pathog. 18, e1010313. doi: 10.1371/journal.ppat.1010313, PMID: 35298557 PMC8929637

[B30] Sibley L. D. Krahenbuhl J. L. Adams G. M. Weidner E. (1986). *Toxoplasma* modifies macrophage phagosomes by secretion of a vesicular network rich in surface proteins. J. Cell Biol. 103, 867–874. doi: 10.1083/jcb.103.3.867, PMID: 3528173 PMC2114290

[B31] Silva V. O. Maia M. M. Torrecilhas A. C. Taniwaki N. N. Namiyama G. M. Oliveira K. C. . (2018). Extracellular vesicles isolated from *Toxoplasma gondii* induce host immune response. Parasite Immunol. 40, e12571. doi: 10.1111/pim.12571, PMID: 29974519

[B32] Skotland T. Ekroos K. Llorente A. Sandvig K. (2025). Quantitative lipid analysis of extracellular vesicle preparations: A perspective. J. Extracell Vesicles 14, e70049. doi: 10.1002/jev2.70049, PMID: 40091364 PMC11911390

[B33] Skotland T. Sagini K. Sandvig K. Llorente A. (2020). An emerging focus on lipids in extracellular vesicles. Adv Drug Deliver Rev. 159, 308–321. doi: 10.1016/j.addr.2020.03.002, PMID: 32151658

[B34] Subramanian A. Tamayo P. Mootha V. K. Mukherjee S. Ebert B. L. Gillette M. A. . (2005). Gene set enrichment analysis: A knowledge-based approach for interpreting genome-wide expression profiles. Proc. Natl. Acad. Sci. 102, 15545–15550. doi: 10.1073/pnas.0506580102, PMID: 16199517 PMC1239896

[B35] Tkach M. Théry C. (2016). Communication by extracellular vesicles: Where we are and where we need to go. Cell 164, 1226–1232. doi: 10.1016/j.cell.2016.01.043, PMID: 26967288

[B36] Torres R. M. Cyster J. G. (2023). Lipid mediators in the regulation of innate and adaptive immunity. Immunol. Rev. 317, 4–7. doi: 10.1111/imr.13228, PMID: 37243330

[B37] Tsugawa H. Cajka T. Kind T. Ma Y. Higgins B. Ikeda K. . (2015). MS-DIAL: Data-independent MS/MS deconvolution for comprehensive metabolome analysis. Nat. Methods 12, 523–526. doi: 10.1038/nmeth.3393, PMID: 25938372 PMC4449330

[B38] van Meer G. Voelker D. R. Feigenson G. W. (2008). Membrane lipids: Where they are and how they behave. Nat. Rev. Mol. Cell Biol. 9, 112–124. doi: 10.1038/nrm2330, PMID: 18216768 PMC2642958

[B39] van Niel G. Carter D. R. F. Clayton A. Lambert D. W. Raposo G. Vader P. (2022). Challenges and directions in studying cell–cell communication by extracellular vesicles. Nat. Rev. Mol. Cell Biol. 23, 369–382. doi: 10.1038/s41580-022-00460-3, PMID: 35260831

[B40] Walsh D. Katris N. J. Sheiner L. Botté C. Y. (2022). *Toxoplasma* metabolic flexibility in different growth conditions. Trends Parasitol. 38, 775–790. doi: 10.1016/j.pt.2022.06.001, PMID: 35718642 PMC10506913

[B41] Walther T. C. Farese R. V. (2012). Lipid droplets and cellular lipid metabolism. Annu. Rev. Biochem. 81, 687–714. doi: 10.1146/annurev-biochem-061009-102430, PMID: 22524315 PMC3767414

[B42] Wang J. Chen Y.-L. Li Y.-K. Chen D.-K. He J.-F. Yao N. (2021). Functions of sphingolipids in pathogenesis during host–pathogen interactions. Front. Microbiol. 12. doi: 10.3389/fmicb.2021.701041, PMID: 34408731 PMC8366399

[B43] Welsh J. A. Goberdhan D. C. I. O’Driscoll L. Buzas E. I. Blenkiron C. Bussolati B. . (2024). Minimal information for studies of extracellular vesicles (MISEV2023): From basic to advanced approaches. J. Extracell Vesicles 13, e12404. doi: 10.1002/jev2.12404, PMID: 38326288 PMC10850029

[B44] Woolhouse M. E. J. Webster J. P. Domingo E. Charlesworth B. Levin B. R. (2002). Biological and biomedical implications of the co-evolution of pathogens and their hosts. Nat. Genet. 32, 569–577. doi: 10.1038/ng1202-569, PMID: 12457190

[B45] Wowk P. F. Zardo M. L. Miot H. T. Goldenberg S. Carvalho P. C. Mörking P. A. (2017). Proteomic profiling of extracellular vesicles secreted from *Toxoplasma gondii*. Proteomics 17, 1600477. doi: 10.1002/pmic.201600477, PMID: 28643940

[B46] Wymann M. P. Schneiter R. (2008). Lipid signalling in disease. Nat. Rev. Mol. Cell Biol. 9, 162–176. doi: 10.1038/nrm2335, PMID: 18216772

[B47] Xu F. Lu X. Cheng R. Zhu Y. Miao S. Huang Q. . (2020). The influence of exposure to *Toxoplasma gondii* on host lipid metabolism. BMC Infect. Dis. 20, 415. doi: 10.1186/s12879-020-05138-9, PMID: 32539811 PMC7294668

[B48] Yáñez-Mó M. Siljander P.-R.-M. Andreu Z. Bedina Zavec A. Borràs F. E. Buzas E. I. . (2015). Biological properties of extracellular vesicles and their physiological functions. J. Extracell Vesicles 4, 27066. doi: 10.3402/jev.v4.27066, PMID: 25979354 PMC4433489

