# Supplementary figures and images for: Toxoplasma gondii assembles extracellular vesicles with conserved lipid profiles across host cell types
Source: Front Cell Infect Microbiol. 2026 Feb 4;16:1745625. doi: 10.3389/fcimb.2026.1745625 (PMC12913473; doi:10.3389/fcimb.2026.1745625)

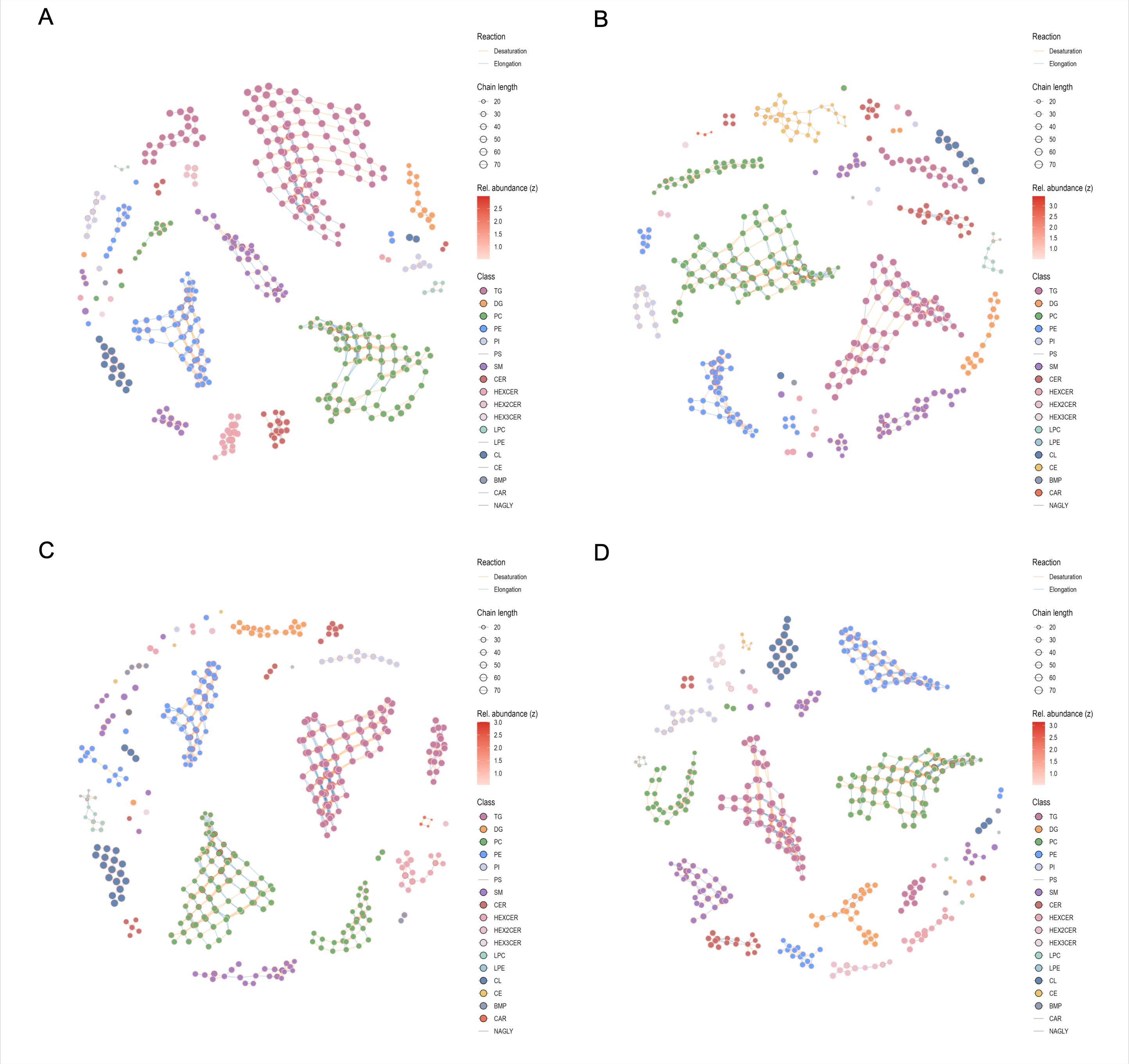

Supplement: Supplementary Figure 1 — Global lipid reaction network topology across four host cell types. (A–D) Lipid reaction networks of the most abundant lipid species in (A) IPEC, (B) fibroblast, (C) myoblast, and (D) Vero cells, reconstructed based on known biosynthetic and degradation pathways. Nodes represent individual lipid species, scaled by carbon chain length and colored by lipid class. Node size indicates relative abundance (z-score normalized), while edges represent enzymatic transformations. Distinct network architectures and dominant lipid clusters highlight cell type–specific lipid metabolic organization. [file Image1.jpeg]

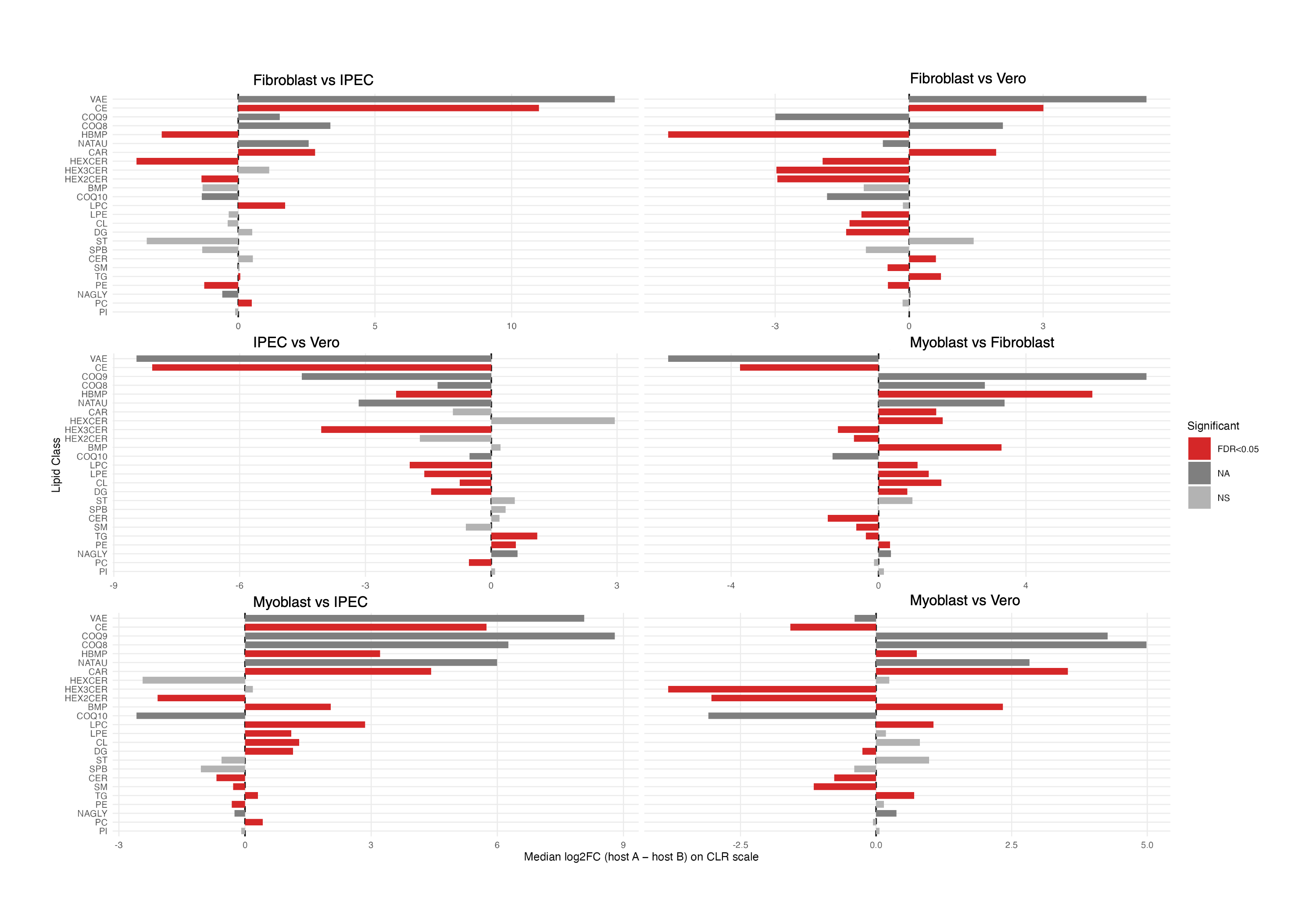

Supplement: Supplementary Figure 2 — Class-level differential lipid abundance between host cell types. Compositional log-ratio (CLR) analysis of lipid class abundance for all pairwise comparisons between host cell types. Bars indicate median log2 fold changes in lipid class abundance (host A – host B). Red bars denote statistically significant differences (FDR < 0.05), grey bars represent non-significant changes (NS), and dark grey indicates classes not testable, not enough lipid species for a statistical test (NA). [file Image2.jpeg]

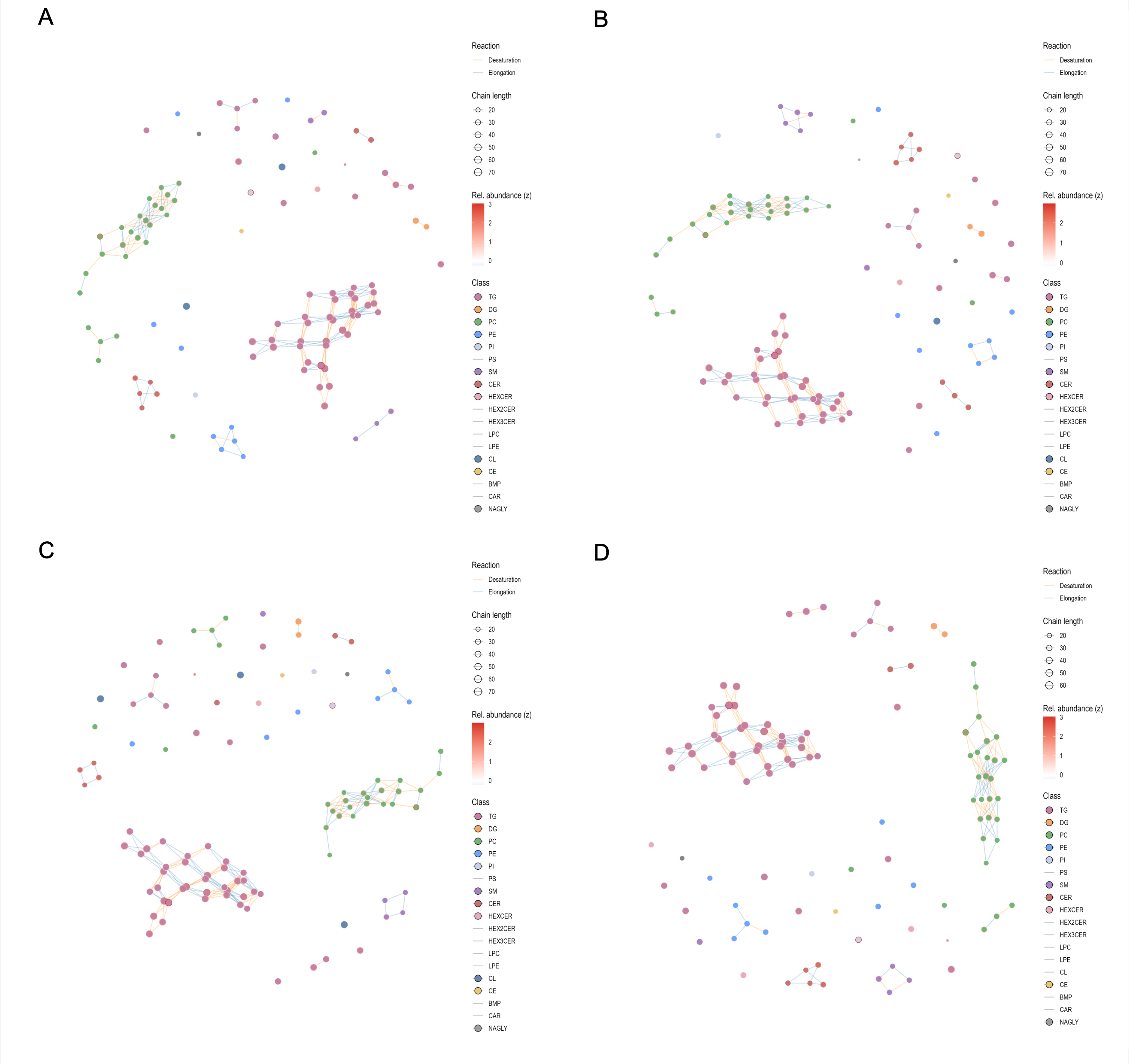

Supplement: Supplementary Figure 3 — Metabolic network topology of TgEV lipidomes. (A–D) Network reconstruction of the most abundant EV lipid species from T. gondii–infected (A) IPEC, (B) fibroblast, (C) Vero, and (D) myoblast. Nodes represent individual lipid species, scaled by chain length and coloured by lipid class. Node size reflects relative abundance (z-score normalized), while edges indicate known enzymatic elongation or desaturation reactions. [file Image3.jpeg]

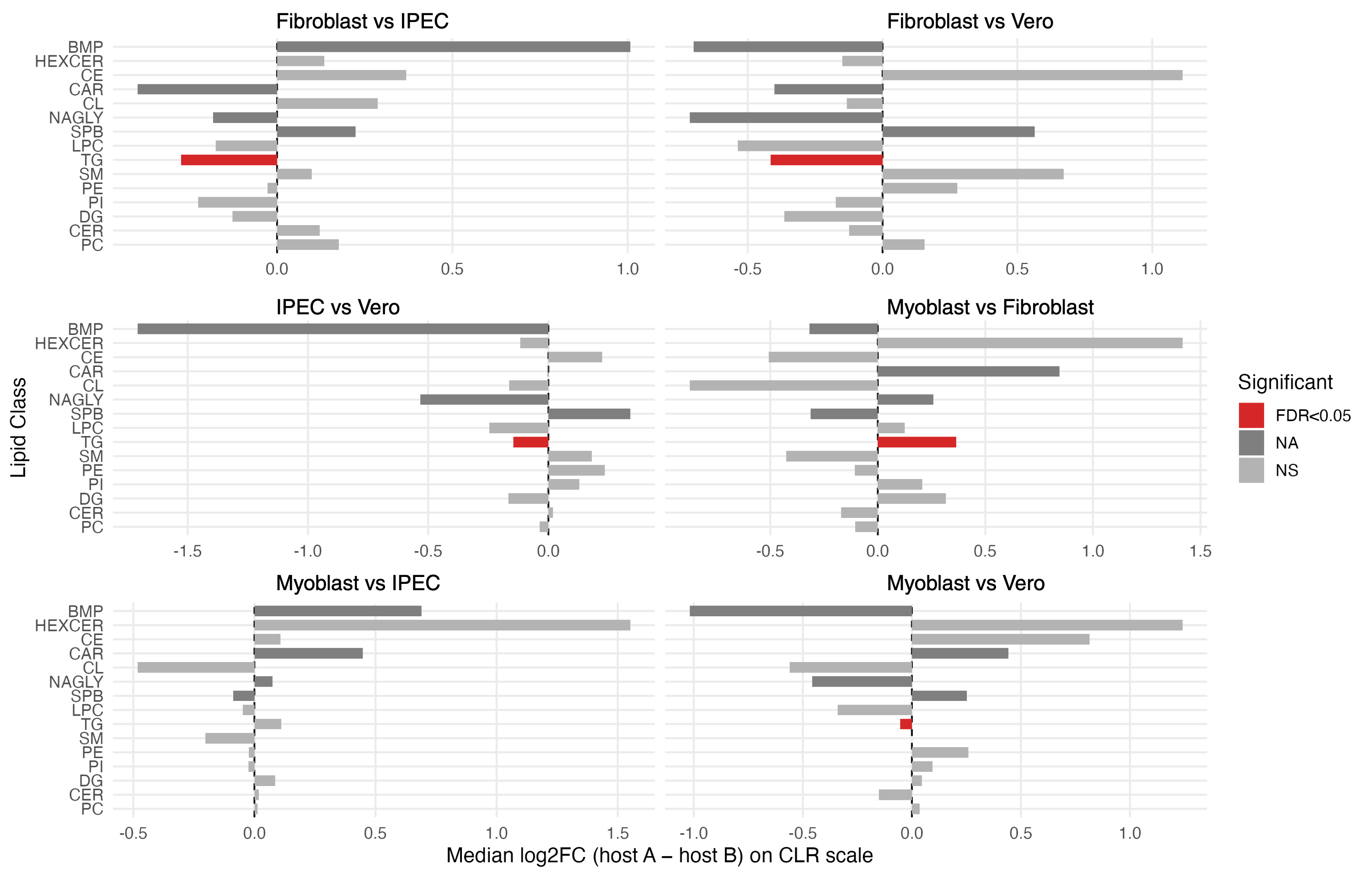

Supplement: Supplementary Figure 4 — Comparative lipid class abundance across TgEVs. Compositional log-ratio (CLR) analysis of lipid class abundances in pairwise comparisons of EV samples from different host cell types. Bars represent median log2 fold changes (host A – host B) for each lipid class. Red bars indicate statistically significant differences (FDR < 0.05), grey bars denote non-significant changes (NS), and dark grey indicates classes not testable (NA). [file Image4.jpeg]

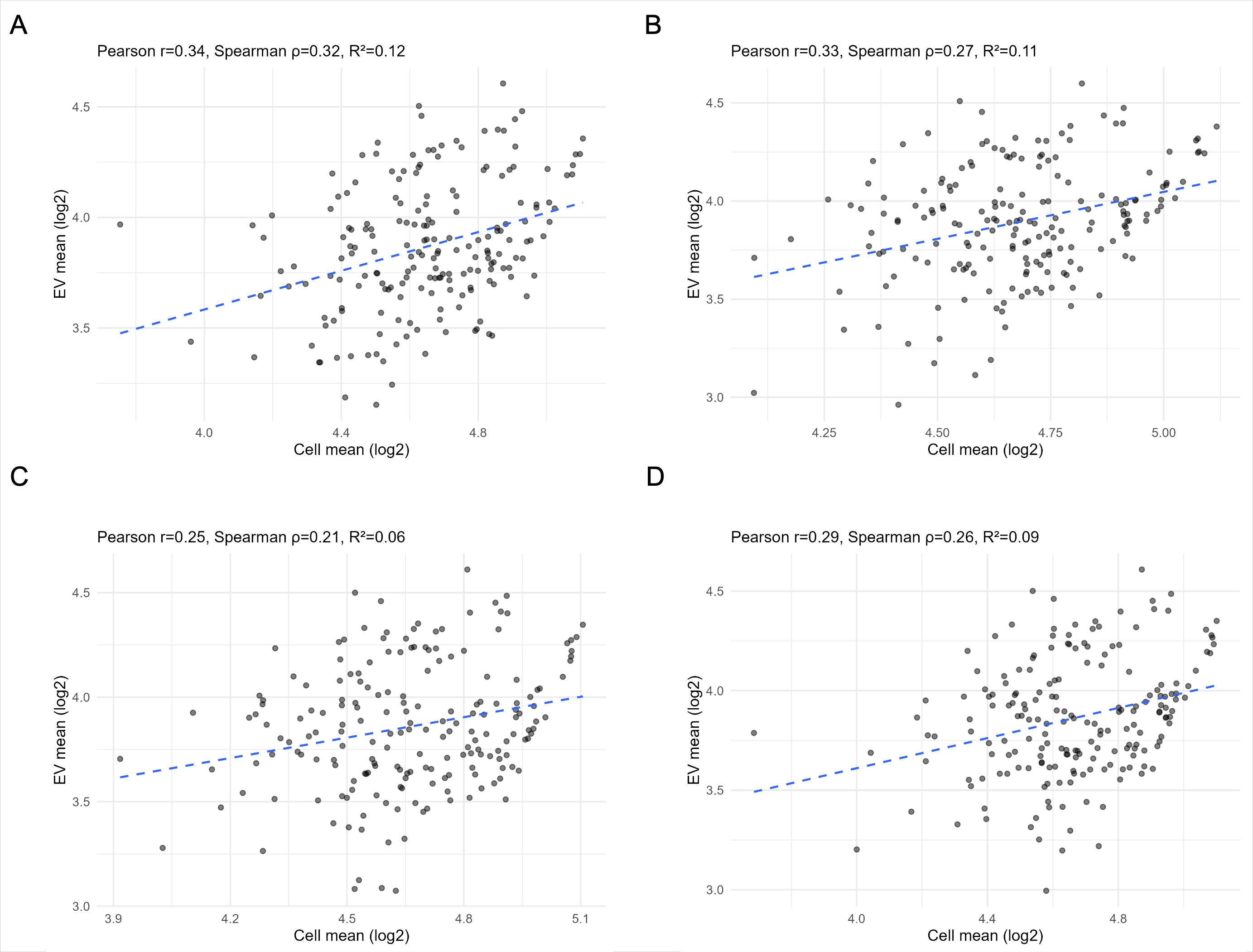

Supplement: Supplementary Figure 5 — Correlation between host cell and T. gondii EV lipidomes reveals moderate overlap and selective lipid sorting. Scatter plots show the correlation of shared lipid species between EVs and host cells for each cell type: (A) IPEC, (B) fibroblast, (C) Vero, and (D) myoblast. Each dot represents the mean log2 abundance of a shared lipid species in the EV (y-axis) versus the cell (x-axis). [file Image5.jpeg]
