# Supplementary material for: Toxoplasma gondii assembles extracellular vesicles with conserved lipid profiles across host cell types
Source: Front Cell Infect Microbiol. 2026 Feb 4;16:1745625. doi: 10.3389/fcimb.2026.1745625 (PMC12913473; doi:10.3389/fcimb.2026.1745625)
Supplement: Supplementary file 9 [file DataSheet1.docx]

**Statistical Analysis of Cellular and Toxoplasma gondii EV Lipidomes**

Lipidomic data from host cells and *T. gondii* extracellular vesicles (TgEVs) were processed and analyzed in R (v4.2.0). Lipid identifiers were standardized according to accepted shorthand notation and annotated by host cell type and material (cell or EV).

**Data preprocessing and normalization.** Background signals were excluded using blank-based filtering, and retained features were required to occur in the majority of replicates within at least one host group. Low-quality samples were identified by total ion current (TIC) and principal component analysis (PCA), and one technical outlier was removed. Intensities were normalized to TIC, log₂-transformed, and missing values imputed using a k-nearest neighbor (KNN) approach.

**Compositional treatment and multivariate analysis.** Given the compositional nature of lipidomic data, analyses were performed on log₂- or centered log-ratio (CLR)–transformed intensities. Global lipidome variation was assessed by PCA, and group-level differences were tested by PERMANOVA using Aitchison distance.

**Differential abundance and comparative analyses.** Differential lipid abundance was assessed using moderated linear models (*limma*) with FDR < 0.05. Fold-change thresholds (|log₂FC| ≥ 0.58 soft, ≥1 reference) were applied for visualization. Relationships between EV and cellular lipidomes were quantified using Pearson correlation and paired differential analysis, with lipid class–level changes evaluated using Wilcoxon tests.

**Enrichment and functional interpretation.** Lipid subclass enrichment was investigated by gene set enrichment analysis (GSEA) based on ranked fold changes, reporting normalized enrichment scores (NES) and FDR-adjusted *p*-values.

Detailed descriptions of quality control, filtering criteria, imputation, compositional transformations, and statistical modeling are provided in the **Supplementary Methods**.

**Outlier detection and removal.** Sample quality was assessed using multiple complementary approaches prior to statistical analysis. We calculated the total ion current (TIC) for each sample and identified potential outliers as those exceeding three median absolute deviations (MAD) from the group median. Principal component analysis (PCA) on z-scored data was used as a second diagnostic, flagging samples with Mahalanobis distances above the χ²₍2,0.001₎ threshold in the PC1–PC2 space. One EV myoblast replicate was excluded on this basis, and raw LC–MS inspection confirmed that this deviation was due to a technical injection failure rather than biological variability.

**Blank Filtering and Feature Retention.** To remove background signals, a blank-based filter was applied on a feature-wise basis. A lipid species was retained if ≥75% of samples in a given material (cell or EV) had intensities above the median blank plus a small offset (ε). The ε parameter was defined as 1×10⁻⁹, a value several orders of magnitude below the smallest non-zero intensity in the dataset, ensuring numerical stability without influencing feature retention. An additional per-host filter required that retained features be present in ≥70% of samples within at least one host group.

**Normalization, Transformation, and Imputation.** To account for differences in sample loading, total ion current (TIC) normalization was applied within each material, scaling sample intensities to the median TIC. Data were then log₂-transformed with a pseudocount (10⁻⁶) to stabilize variance and reduce heteroscedasticity. After TIC scaling and log₂(+pseudocount) transformation, missing values were imputed with k-nearest neighbors (KNN) using impute.knn (R, impute package; default settings, k = 10), applied separately within each material (EVs and Cells) with lipids as rows and samples as columns. For each lipid, missing entries were estimated as a weighted average of the most similar lipids based on their observed profiles. KNN assumes that missingness is approximately missing-at-random conditional on local similarity and typically preserves covariance structure better than single-value fills, which benefits multivariate analyses (e.g., PCA, PERMANOVA). As protection against over-borrowing, features failing the a priori detection criteria were removed before imputation. We note that KNN can shrink extreme values when whole lipid subclasses are sparsely observed; accordingly, statistical inference relies on FDR-controlled linear models, and the imputation choice is reported for transparency.

**Compositional Data Considerations.** Given the compositional nature of lipidomics data, analyses were performed on log₂-transformed data and, where appropriate, on centered log-ratio (CLR)–transformed data. CLR transformation was implemented by subtracting the sample-wise column mean from the log₂-transformed intensities, equivalent to centering by the geometric mean on the original scale. CLR-transformed data were used for compositional analyses, lipid class comparisons, and dissimilarity-based tests.

**Multivariate Analyses and Global Lipidome Differences.** Global differences in lipidome composition between groups were visualized using PCA on z-scored data. Group separation was further tested using permutational multivariate analysis of variance (PERMANOVA, 999 permutations). Because lipidomic data are compositional, Euclidean distance on CLR-transformed data (Aitchison distance) was used as the primary dissimilarity metric. For comparability with previous lipidomics studies, PERMANOVA was also performed on Euclidean distances derived from z-scored data, yielding consistent results. Models included host cell type, material (EV vs. cell), and their interaction as fixed factors.

**Differential Abundance Analysis.** Differential abundance between experimental groups (e.g., across host types within EVs or cells) was assessed using moderated linear models implemented in limma. Empirical Bayes moderation was applied to improve variance estimates, and statistical significance was defined as a Benjamini–Hochberg false discovery rate (FDR) < 0.05. Fold-change thresholds were used solely for visualization and prioritization: |log₂FC| ≥ 0.58 (1.5-fold) as a soft guide and |log₂FC| ≥ 1 as a reference. These cutoffs reflect biologically meaningful shifts for membrane lipids, which often exert significant functional effects with relatively small abundance changes.

**EV–Cell Lipidome Comparisons.** To assess the relationship between EV and cellular lipidomes, analyses were restricted to lipid species detected in both sample types. PCA and hierarchical clustering were used to visualize similarities and differences between matched EV and cell lipidomes, while correlation analysis (Pearson’s r on log₂-transformed means) quantified their overall association. Only weak to moderate correlations (r = 0.21–0.34) were observed, consistent with selective lipid sorting rather than passive inheritance. Paired differential abundance analysis was performed using a duplicateCorrelation model in limma, accounting for matched replicates. Lipid-level differences (EV – cell) were calculated to quantify enrichment or depletion, and per-class median CLR log₂ fold changes were assessed using Wilcoxon signed-rank tests (BH-corrected). Lipid class assignment was based on the first token of the lipid name, which was standardized and manually verified; rare ambiguities (<1%) were resolved by chain-length inspection.

**Gene Set Enrichment Analysis (GSEA).** To evaluate systematic enrichment of lipid subclasses, GSEA was performed using ranked log₂ fold changes from limma contrasts. Subclass gene sets were derived from the lipid shorthand notation, and normalized enrichment scores (NES) and FDR-adjusted p-values were reported. Positive NES values indicate enrichment in EVs relative to cells, while negative NES values indicate depletion.
